# Developmental divergence in gene regulation underlies trophic innovation in Lake Victoria cichlids

**DOI:** 10.1098/rspb.2025.3318

**Published:** 2026-10-07

**Authors:** Anna Duenser, Pooja Singh, Ehsan P. Ahi, Marija Durdevic, Sylvia Schäffer, Julian Gallaun, Wolfgang Gessl, Ole Seehausen, Christian Sturmbauer

**Affiliations:** ^1^ Institute of Biology, University of Graz, Graz, Austria; ^2^ Institute for Aquatic Ecology and Fisheries Management, Federal Agency for Water Management, Austria; ^3^ Institute of Ecology and Evolution, University of Bern, Bern, Switzerland; ^4^ Swiss Federal Institute of Aquatic Science and Technology (EAWAG), Kastanienbaum, Lucerne, Switzerland; ^5^ Organismal and Evolutionary Biology Research Programme, University of Helsinki, Helsinki, Uusimaa, Finland; ^6^ Diagnostic and Research Centre for Molecular BioMedicine, Institute of Pathology, Medical University of Graz, Graz, Austria; ^7^ Core Facility Computational Bioanalytics, Centre for Medical Research, Medical University of Graz, Graz, Austria

**Keywords:** adaptation, evolution, gene expression, alternative splicing, adaptive radiation

## Abstract

The role of developmental shifts in the emergence of ecological diversity during adaptive radiation remains largely uncharacterized. Here, we investigate the developmental gene regulatory landscape of trophic innovation in the rapid adaptive radiation of cichlid fishes from Lake Victoria (LV), which evolved into hundreds of species in less than 16 000 years. By analysing the whole transcriptomes of functionally decoupled oral and pharyngeal jaws from two life stages, we show that both gene and isoform expression are highly developmentally dynamic in ecologically divergent species. Gene regulation is conserved at the tissue level in the jaws of ecologically distinct species at the early life stage and then diverges into guild-specific developmental programmes in adults. We further show that gene expression and alternative splicing regulate different pathways, particularly in adults, suggesting that these two gene regulatory mechanisms are constrained to perform non-redundant functions, at least at the early stages of adaptive radiation. Interestingly, we found that splicing-mediated exonization of the craniofacial development gene, *kaznb*, may have contributed to the evolution of herbivory in LV cichlids. Overall, our study suggests that ontogenetic shifts in gene regulation play a key role in the rapid emergence of guild-specific ecological diversity in LV cichlids.

## 1. Introduction

Since King & Wilson [1] concluded that variation in protein sequence alone cannot explain the phenotypic differences between humans and chimpanzees, transcriptional regulation has been shown to play a fundamental role in phenotypic evolution and diversification [2–4]. However, few studies have focused on rapid adaptive radiations [5–9], where species diversify rapidly in tight connection with ecology, and thus, regulatory changes are hypothesized to dominate phenotypic divergence, as they can emerge more rapidly from standing genetic variation and relaxed selection than de novo protein-coding variation. Furthermore, little is known about gene regulatory changes during development of key innovations in adaptive radiations in multi-species comparisons, and this has only recently begun to be explored in model species [10].

Cases of adaptive radiation provide promising model systems to study the role of gene regulation in rapid evolutionary diversification [11]. In the adaptive radiations of cichlid fishes in the Great East African lakes, rapid diversification proceeded along many ecological and morphological axes (reviewed in [12]). The Lake Victoria (LV) cichlid radiation comprising approximately 500 species is arguably one of the most spectacular of these radiations, having emerged after the complete desiccation of the lake less than 16 000 years ago [13]. Entire food webs have evolved in this radiation, with species adapted to diverse herbivorous and carnivorous trophic niches [14]. The functionally decoupled oral and pharyngeal jaws of cichlids are hypothesized to be a key innovation and the basis of their evolutionary success that enabled rapid trophic diversification [15].

Ontogenetic diet shifts are common in animals [16]. Theory suggests that they can alter the trajectory of adaptive radiation in the presence of ecological opportunities [17]. However, little attention has been directed towards the role of ontogenetic developmental changes during adaptive radiation [18]. In cichlids, there is some evidence that key shape changes in cichlid jaw cartilage and bone occur during early development [19] and that specialized morphologies are already distinct between trophic niches at the onset of exogenous feeding at stage 26 [4,7]. But most transcriptional studies across multiple species have focused on single developmental stages [6,7,20] (but see [21]), and we understand little about transcriptional mechanisms underlying the development of diverse adaptive trophic morphologies.

Here, we leveraged a comparative framework to investigate the differences and commonalities between two postembryonic life stages in cichlid species from LV: the end of the larval and beginning of the juvenile stage stage 26 [22] and the adult stage. The end of the larval development (stage 26) is marked by the complete absorption of the yolk sac and denotes the onset of independent foraging [22]. The adult stage (marked by the onset of breeding and the emergence of male nuptial colouration [23]) represents full maturity of the entire phenotype. To identify developmental genes and regulatory mechanisms involved in trophic differentiation, we explored the landscape of gene expression and alternative splicing underlying the trophic adaptations of the oral jaw apparatus (OJA) and pharyngeal jaw apparatus (PJA) (figure 1C,D) in LV cichlids (figure 1).

**Figure 1.**
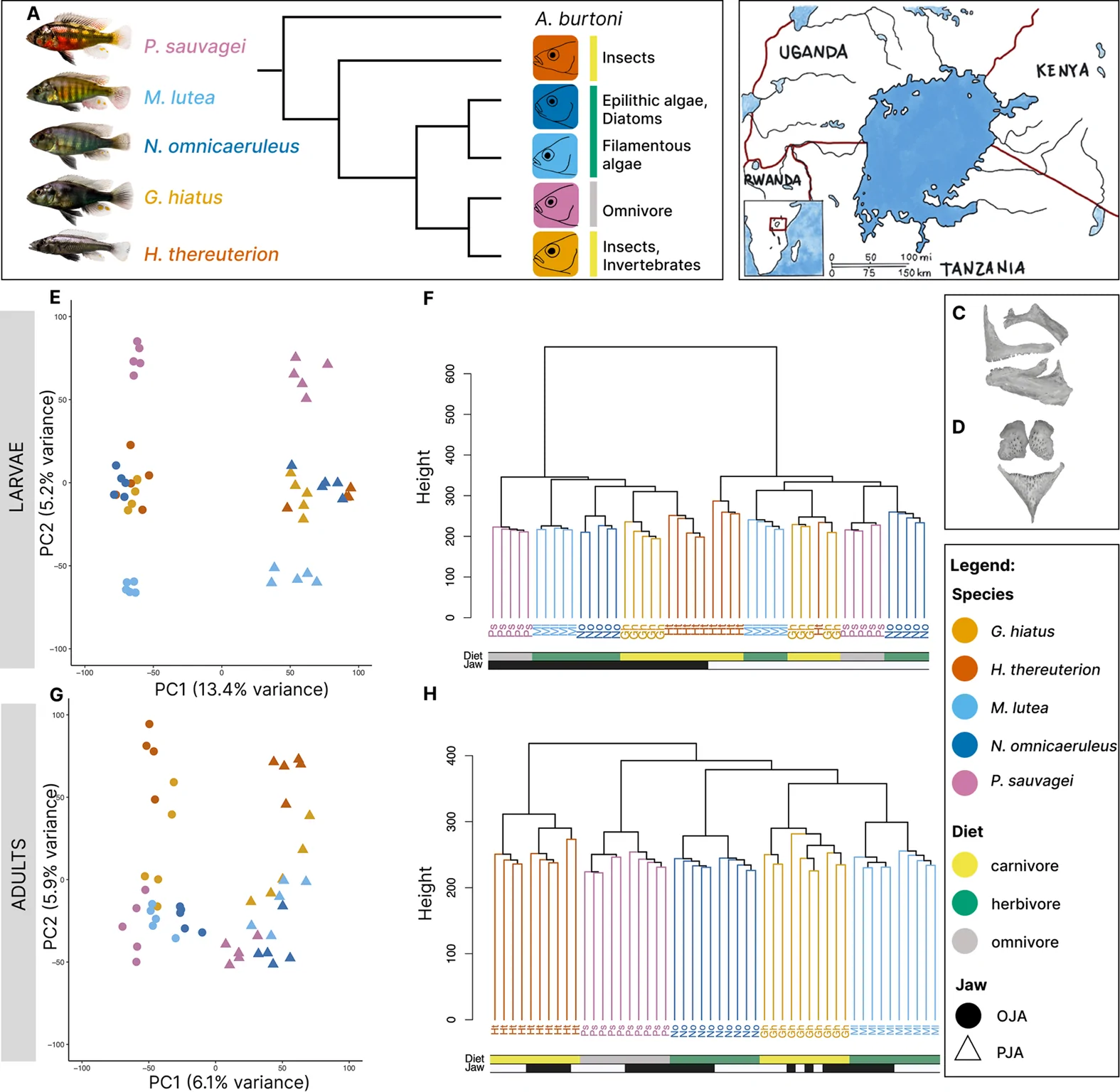
Overview of the study design illustrating LV species, their trophic specializations and broad patterns of gene expression. (A) Study species, their respective trophic niches and cladogram of species relationships adapted from Meier *et al.* [14] with *Astatotilapia burtoni* included as outgroup. These species represent the evolution of herbivory and carnivory in the LV radiation (Meier *et al.* [14] shows genome-wide phylogeny of all sequenced species from this radiation). (B) Map of LV in East Africa. (C) OJA. (D) PJA. Transcriptome-wide gene expression results across two developmental stages were investigated for each species: larvae (developmental stage 26 [22]) and adults (onset of sexual maturity; males). Normalized gene expression of 37 334 genes was used for PCA plots for (E) larvae, (G) adults, (F) dendrograms and (H) of the Euclidean distance. Dendrogram annotations: OJA (black) and PJA (white). PCA annotations: OJA; circles and PJA; triangles. Abbreviations: *G. hiatus* (Gh; bright orange; carnivore), *H. thereuterion* (Ht; dark orange; carnivore), *M. lutea* (Ml; bright blue; herbivore), *N. omnicaeruleus* (No; dark blue; herbivore) and *P. sauvagei* (Ps; pink; omnivore). Results for isoform expression are shown in figure S2 in the supplementary material.

## 2. Results

### (a) Species selection and genome-guided transcriptome assembly and annotation

To analyse the transcriptional landscape of LV cichlids adapted to different diets, we generated whole mRNA transcriptomes from the OJA and PJA of five species: *Gaurochromis hiatus* (Gh), *Haplochromis thereuterion* (Ht), *Mbipia lutea* (Ml), *Neochromis omnicaeruleus* (No) and *Paralabidochromis sauvagei* (Ps). These five species represent herbivorous and carnivorous trophic adaptations in the LV cichlid adaptive radiation [14]. Compared with herbivores, carnivores have longer, shallower skulls and longer upturned oral jaws, whereas herbivores have shorter, deeper skulls, shorter downturned oral jaws, deeper pharyngeal keels, molarized teeth (supplementary material, figure S1), and more oral tooth rows for algae scraping [23,24]. All species were sampled across two developmental stages (larval stage 26–yolk sac absorption into body cavity and adulthood–onset of male nuptial colouration; figure 1). Each stage, species and jaw was represented by five biological replicates. Tissues from larvae were pooled from two individuals for each biological replicate owing to their small size. Our total sample set comprised 100 whole transcriptomes (140 samples including the outgroup cichlid species *Tropheops tropheops* from Lake Malawi and an East African riverine species *Astatotilapia burtoni*). After quality filtering, we retained an average of 3–9 million reads per sample. Of these reads, 68–82% could be mapped to the reference genome of *Oreochromis niloticus* [25] in the larval samples and 75–82% in the adult samples (supplementary material, file 1). We chose to map to the *O. niloticus* reference genome because it is the best-annotated reference genome and high-quality transcript annotations are important for gene regulation analyses. The final merged annotation of all samples included 37 334 genes and the transcript annotation 59 849 transcripts.

### (b) Transcriptome-wide patterns of gene and isoform expression

We quantified the expression of 37 334 genes across all samples. To obtain an overview of global gene expression patterns, we conducted a principal component analysis (PCA) based on normalized expression values. The PCA showed a clear separation of expression profiles of the two jaw tissue sets for both life stages, but the separation of the two jaws was less prominent in adults (larvae: PC1 13.4%; adults: PC1 6.1%; figure 1E,G). We also quantified expression of 59 849 transcripts to see how isoform expression compared to gene expression. The larval isoform expression PCA (supplementary material, figure S2A) showed similar patterns to the larval gene expression profiles (figure 2A), with a clear distinction between the two jaw apparatuses but less differentiation among species (PC1 11.4%)). Isoform expression clustering in adults (supplementary material, figure S2C) was more guild-specific, but less variation was explained by PC1 (5.6%) and PC2 (4.4%) when compared to the gene expression PCA (figure 1C).

**Figure 2.**
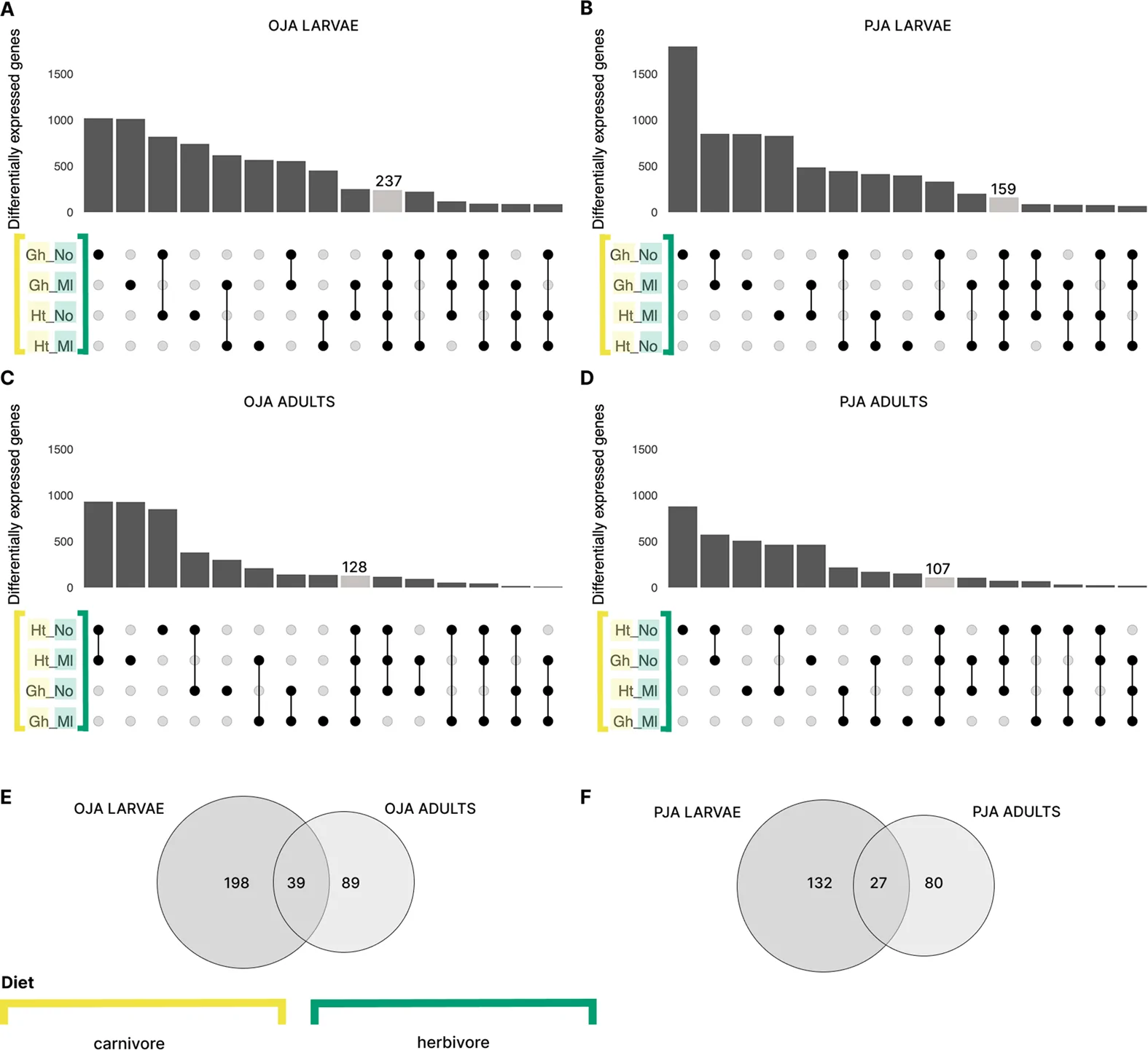
Differentially expressed genes between herbivorous and carnivorous LV cichlids from pairwise species comparisons. UpSet plots show numbers of differentially expressed genes per comparison and overlaps among comparisons; yellow and green brackets mark carnivorous and herbivorous species, respectively. Points below bars indicate shared or comparison-specific gene sets, and light grey bars mark genes shared across all pairwise comparisons. UpSet plots are shown for (A) larval OJA, (B) larval PJA, (C) adult OJA and (D) adult PJA. Venn diagrams show overlap of shared differentially expressed genes between larvae and adults for (E) OJA and (F) PJA. Abbreviations: *G. hiatus* (Gh; carnivore), *H. thereuterion* (Ht; carnivore), *M. lutea* (Ml; herbivore) and *N. omnicaeruleus* (No; herbivore).

Hierarchical clustering of expression data showed distinct patterns across stages (figure 1F,H). In larvae, OJA and PJA clustered separately; OJA followed the trophic niche, whereas PJA clustered by species (figure 1F). In adults, clustering was strictly guild-specific, regardless of jaw tissue (figure 1H). Isoform expression clustering largely matched gene-expression patterns, except for slight differences in larval PJA species clustering (supplementary material, figure S2B,D).

To place expression patterns into a wider phylogenetic context, we added *T. tropheops* and *A. burtoni* as divergent outgroups from Lake Malawi and rivers around Lake Tanganyika, respectively. The PCA as well as the hierarchical cluster analysis based on Euclidean distance showed a clear separation of expression profiles of the outgroup from the LV species (supplementary material, figure S3).

### (c) Genes involved in species and trophic differentiation in Lake Victoria cichlids

To identify genes that contribute to species differences in oral and pharyngeal jaws of LV cichlids, we conducted differential expression analysis for all pairwise species comparisons for each jaw and developmental stage. Of the 37 334 genes, 5743–10 024 (15.4–26.9%) showed significant (adjusted *p*-value < 0.05) differential expression between at least one pairwise species comparison in all tissues and at both developmental stages (supplementary material, table S1). Interestingly, no gene was differentially expressed across all comparisons of species and tissues. To assess the relative contribution to gene expression variation of species/guild identity versus trophic niche, we included a variance partitioning analysis. While this analysis should be regarded with some caution (only five species and with trophic niche being a species-level trait), it showed that species/guild identity explains more variation than trophic niche (mean percentage adult: species: 7.76%, diet: 4.79%, larvae: species: 11.47% and diet: 3.14%).

To identify genes that may play a role in trophic adaptation of the jaw apparatuses beyond the backdrop of species or guild identity, we primarily focused on pairwise comparisons of carnivorous species (Ht and Gh) to the herbivorous species (Ml and No). We identified the intersection set of genes that were differentially expressed across all four pairwise comparisons (Gh versus Ml, Gh versus No, Ht versus Ml and Ht versus No) as genes that are important for differentiating the jaws of herbivores from those of carnivores (figure 2). We discovered 237 genes that were differentially expressed in larval OJA across all four comparisons, and 159 genes were differentially expressed in the larval PJA across all four comparisons (figure 2A,B, respectively). In adult OJA, we identified an overlap of 128 genes that were consistently differentially expressed across all four comparisons and 107 genes were differentially expressed in the PJA across all four comparisons (figure 2C,D). Of these gene sets 79–96% shared the same direction of differential expression in all stages and jaws between carnivores and herbivores (supplementary material, table S2). Such genes that were upregulated in carnivorous species and downregulated in herbivorous species, or vice versa, are candidate genes for divergent trophic adaptation in cichlid fishes. We conducted a signed Stouffer analysis (FDR < 0.05), which gives a more sensitive extension to our strict approach. This also confirmed the majority of our genes (adults: OJA: 96.67%, PJA: 95.15%; larvae: OJA: 69.12%, PJA: 71.81%).

For each jaw tissue and developmental stage, we generated hierarchically clustered heatmaps of shared genes that were differentially expressed in all carnivore-herbivore pairwise comparisons. (grey bars in figure 2A–D). We also included the expression of these genes from the omnivorous LV species *P. sauvagei*. The heatmap clustering placed *P. sauvagei* closer to the carnivorous *G. hiatus* and *H. thereuterion* for each jaw and stage (supplementary material, figure S4A‒D). Additionally, we conducted Gene Ontology (GO) enrichment analysis to identify the biological processes that these genes were involved in. We found that larval OJA genes were associated with jaw development related terms such as ‘roof of mouth development’ (*sox9b*) and ‘collagen catabolic process’ (*mmp2*); and also more general functions like ‘netrin-activated signalling pathway’ involved in axon extension and cell migration, ‘regulation of lipid catabolic process’ and ‘actin filament severing’ (figure 3C, full list: supplementary material, file 2). Genes differentially expressed in the larval PJA showed enrichment for GO terms involved in functions like ‘regulation of cell shape’ (*stat3* and *amotl2b*) and ‘skeletal muscle satellite cell migration’ (*rhocb*) (supplementary material, file 2). In adult OJA, genes were significantly associated with the ‘positive regulation of canonical Wnt signalling pathway’ (*bambia* and *fam53b*) as well as ‘Wnt signalling pathway’ (*bambia*, *bcl9l* and *fam53b*). Interestingly, genes differentially expressed in the adult PJA showed a significant enrichment in ‘regulation of alternative mRNA splicing, via spliceosome’ (*rbfox1*) (supplementary material, file 2).

**Figure 3.**
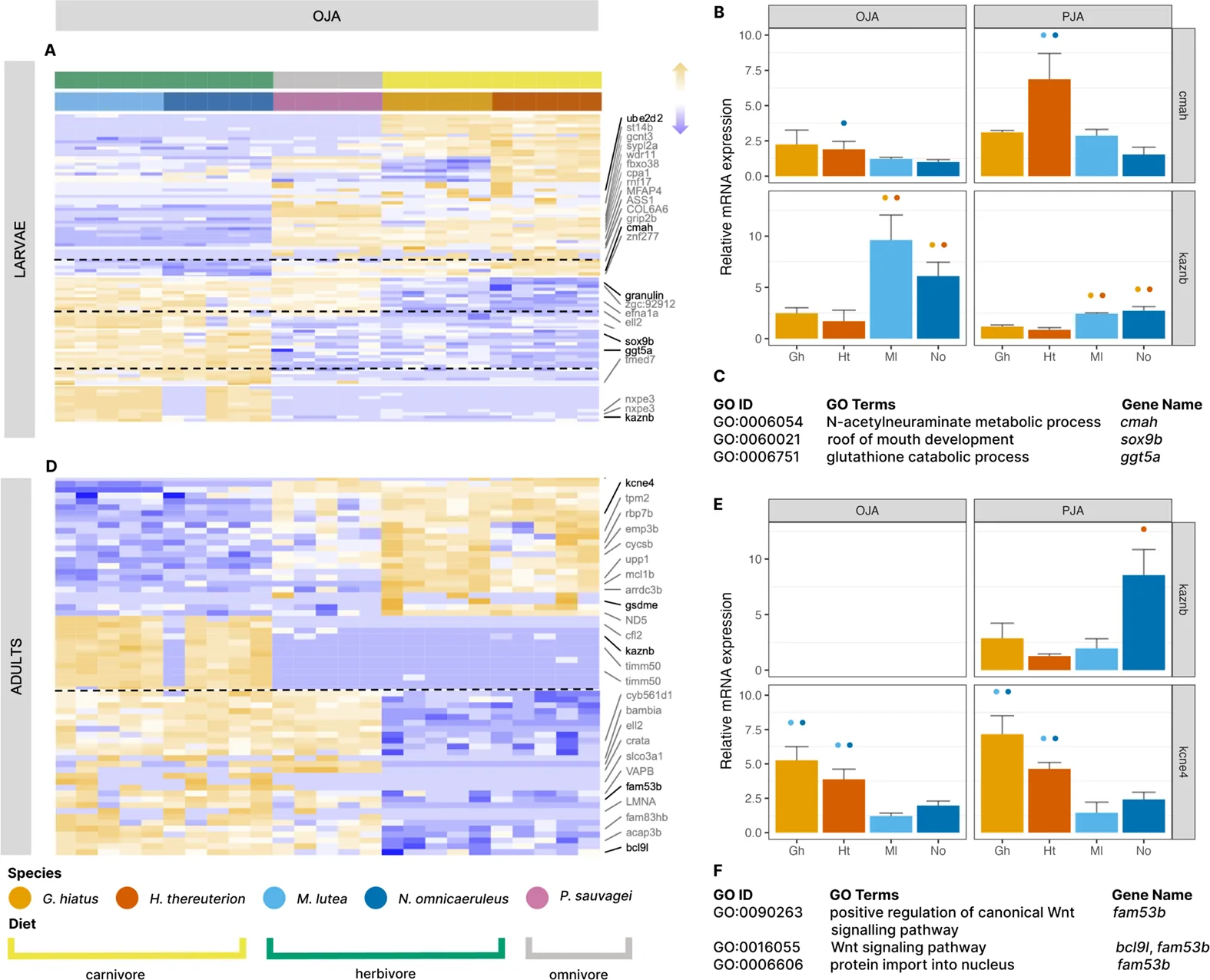
Candidate genes associated with herbivorous and carnivorous jaw adaptations in LV cichlids. Genes shown were consistently differentially expressed in all pairwise comparisons from figure 2 and in the same direction, either higher in carnivores/lower in herbivores or vice versa. Heatmaps show downregulated genes in blue and upregulated genes in orange, with dashed lines separating expression-direction subsets for (A) larval OJA and (D) adult OJA; PJA heatmaps and full OJA heatmaps are in figure S4A‒D in the supplementary material. Bold gene names mark jaw development candidates used for qPCR validation and/or enriched for development or morphology GO terms. Relevant GO terms are shown for (C) larval OJA and (F) adult OJA. Bar plots show qPCR validation of (B) *cmah* and *kaznb* in larvae and (E) *kaznb* and *kcne4* in adults. Coloured dots indicate significant species differences. Abbreviations: *G. hiatus* (Gh; bright orange; carnivore), *H. thereuterion* (Ht; dark orange; carnivore), *M. lutea* (Ml; bright blue; herbivore), *N. omnicaeruleus* (No; dark blue; herbivore) and *P. sauvagei* (Ps; pink; omnivore).

### (d) Differentially expressed genes spanning both life stages

Comparing shared carnivore-herbivore differentially expressed gene sets between both life stages showed an overlap of 39 genes in the OJA (from 237 larval and 128 adult OJ genes; figure 2E) and 27 genes in the PJA (from 159 larval and 107 adult PJ genes; figure 2F). In the OJA, 25 genes were downregulated and 13 were upregulated in carnivores relative to herbivores in both stages. In the PJA, 18 genes were downregulated and 8 were upregulated in both stages. One gene in each jaw differed in the direction of expression between the stages (supplementary material, file 3).

One gene that was consistently downregulated in the two carnivorous species compared to the herbivorous species in both tissues and stages was *kaznb*, also called *kazrin* o*r periplakin-interacting protein b* (figure 3A,D). We validated *kaznb* expression using qPCR analyses and found that it largely supports the RNA-seq results (figure 3B,E; supplementary material, figure S5 for full results). An inspection of the *kaznb* gene annotation in LV cichlids revealed that the differentially expressed transcript of interest is absent in the reference *O. niloticus* genome annotation. This transcript spans an intronic region in the *O. niloticus* reference and is not linked to any other annotated exons of *kaznb*. This suggests that an intron may have been exonized, through which both novel coding variation (new exon) and novel regulatory variation (new mRNA isoform) may have evolved in the ancestor of LV cichlids or in the course of adaptive radiation within the lake. Interestingly, the exonized sequence maps to a transposable element in the NCBI database.

We additionally compared gene expression between oral and pharyngeal jaws in herbivorous and carnivorous species across life stages to test for trophic differences in jaw regulatory architecture. Although far more genes differed between jaws in larvae than in adults, a conserved core of 121 genes showed consistent jaw-specific expression across all species and stages and was enriched for cranial skeletal development and transcriptional regulation, including *Hox* genes and *alx3* (supplementary material, figure S11).

### (e) Differential alternative splicing underlying trophic adaptation

To investigate the role of alternative splicing in trophic adaptation, we conducted pairwise species comparisons between herbivorous and carnivorous species to detect differential transcript usage, which is a proxy for alternative splicing [26]. The number of differentially used isoforms ranged from 671 to 980 across all comparisons at each stage in both tissues (supplementary material, table S3). A much smaller number of isoforms (than genes) were significantly differentially used across all pairwise comparisons (supplementary material, figure S6). In total, we found 56 isoforms to be differentially used between carnivorous and herbivorous species in the OJA at the larval stage and 43 isoforms in the PJA at the larval stage (supplementary material, figure S6A,B). At the adult stage, we found 75 isoforms in the OJA to be differentially used and 78 isoforms in the PJA at the adult stage (supplementary material, figure S6C,D). The intersection sets of differentially used isoforms between the stages per tissue revealed an even smaller overlap (compared to gene expression analysis) with 20 isoforms between the OJA of larvae and adults and seven between the PJA (supplementary material, figure S6E,F).

To visualize differential isoform usage, we used the median PSI (percentage or proportion spliced in) values [26] that describe the relative abundance of each isoform for differentially spliced transcripts between the carnivorous and herbivorous species comparisons (supplementary material, figure S7). Two isoforms of *fgfr2* (fibroblast growth factor receptor 2) were differentially used in the OJA (figure 4A,D) at both stages and in the PJA of adults (supplementary material, figure S7) but showed a trophic signal only in the larval OJA tissue. *Fgfr2 isoform 1 (fgfr2.1)* was expressed only in the herbivores, and isoforms 1 and 2 *(fgfr2.1* and *fgfr2.2)* were expressed in the carnivores. In the adult OJA, a gene called *frzb* (frizzled-related protein) showed a clear and consistent distinction between the carnivorous and herbivorous species (figure 4E). In the herbivores, only one isoform was used (*frzb.1*), while in the OJA of the adult carnivorous and omnivorous species, the other isoform (*frzb.2*) showed a higher median PSI value (figure 4E). *Frzb* was also significantly differentially used in our comparisons of larval OJA tissue and was identified in the GO enrichment analysis of differentially used transcripts. However, the PSI pattern of this gene is more guild-specific than trophic niche-specific at the larval stage, with both isoforms only expressed in Ht (carnivorous) OJA tissue. A gene that showed very similar patterns in the OJA tissues at both stages and in the adult PJA is *col6a1*, collagen type VI alpha 1 chain, associated with maintenance of tissue integrity, including bone and cartilage [27]. The two isoforms of *col6a1* were used equally in the carnivorous species, while in the herbivores *col6a1.1* isoform showed a higher rate of usage than *col6a1.2* (figure 4C,F). The PJA of larvae showed the most dynamic pattern of isoform usage compared to the PJA of adults as well as the OJA at both stages (supplementary material, figure S7B).

**Figure 4.**
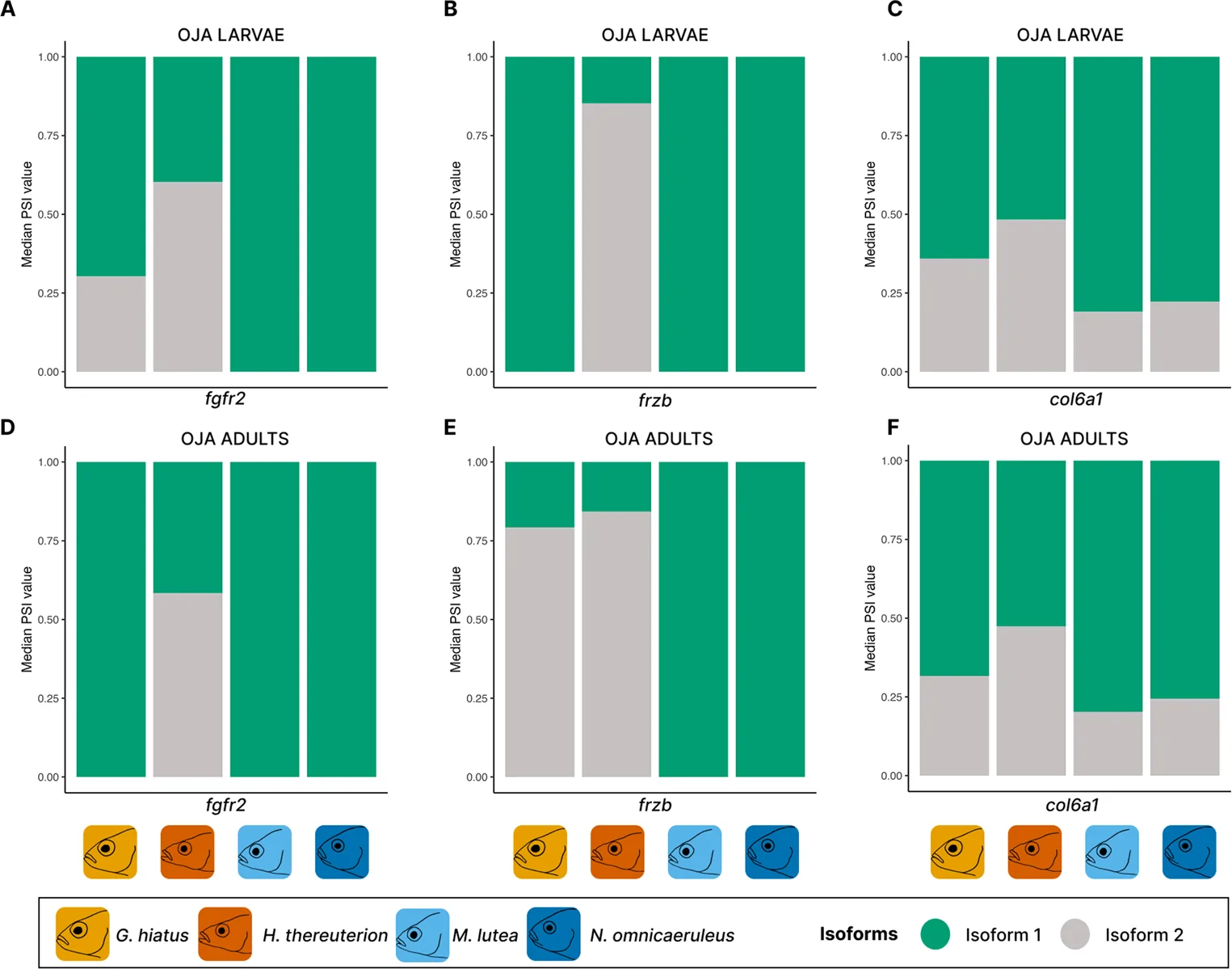
Alternative isoform expression of jaw-development candidate genes in trophically divergent LV cichlids. Median PSI values are shown for isoforms significantly differentially used in carnivore-herbivore pairwise comparisons in (A–C) larval and (D–F) adult OJA. PSI measures each isoform's expression relative to total gene expression [26]. Three genes are shown: *fgfr2*, *frzb* and *col6a1*, each with two isoforms. Abbreviations: G. hiatus (bright orange; carnivore), H. thereuterion (Ht; dark orange; carnivore), M. lutea (bright blue; herbivore), N. omnicaeruleus (dark blue; herbivore) and P. sauvagei (pink; omnivore).

GO enrichment analysis of genes that had significantly different transcript usage showed *frzb* and *fgfr2* to be significantly enriched in the OJA at both stages, as well as the PJA of adults (supplementary material, file 5). *Frzb* was associated with ‘chondrocyte proliferation’ and *fgfr2* with ‘fibroblast growth factor receptor signalling pathway’ in larvae and ‘chondrocyte proliferation’, ‘peptidyl-tyrosine phosphorylation’ and ‘peptidyl-tyrosine modification’ in the adult OJA, as well as ‘positive regulation of cell proliferation’ and ‘transmembrane receptor protein tyrosine’ in adult PJA GO enrichment analysis.

### (f) Contrasting roles for differential gene expression and alternative splicing

To investigate if differentially expressed genes were also alternatively spliced, we looked at the intersection sets of both analyses. Interestingly, there was minimal overlap (supplementary material, figure S8): only one gene was both differentially expressed as well as differentially spliced in the OJA of larvae but was not annotated with a gene name (supplementary material, figure S8). Enriched GO terms also showed little to no overlap (supplementary material, file 2 and 5, figure S9). This suggests that gene expression and alternative splicing perform non-redundant functions during morphogenesis and development.

## 3. Discussion

Rapid changes in how genes are regulated may be an important driver of adaptive evolution at short time scales and during the early stages of speciation [4]. While the role of gene regulation has been studied in the context of adaptation [4–7,28,29] its developmental and ontogenetic dynamics are not well understood. We studied the extremely young adaptive radiation of cichlid fishes from LV and found evidence for dynamic developmental shifts in how genes are expressed and spliced, which may underlie the emergence of their different ecological and morphological adaptations.

### (a) Transcriptional dynamics of cichlid jaw development

Studying natural populations that have undergone adaptive radiation can provide insights into how organisms adapt to changing environments and the genetic and molecular mechanisms behind these processes. We analysed the transcriptional landscape of oral and pharyngeal jaws in LV cichlids and found largely conserved signatures of gene and isoform expression at the tissue level at the early larval developmental stage, but this changed dramatically at the adult life stage where gene and isoform expression became guild-specific (figure 1; supplementary material, figure S2). This indicates that gene regulation is re-wired during development to produce specialized adult morphologies. Clustering of samples within the OJA was, however, already trophic-niche-specific at the larval stage, unlike that of the PJA. This may suggest the importance of the early development of prey capturing versus processing in preparation for when the larvae leave the mother's mouth to start feeding independently. This is consistent with earlier findings that show that craniofacial shape at the larval stage 26 reflects the trophic ecology of species [7,19]. Pharyngeal jaws are used for processing food and may only differentiate between the post-larval and adult stages, as cichlid larvae for all species most likely start off feeding on zooplankton when they are small and constrained by body size, before they switch to their specialized diets as adults [30]. Our analyses of differentially expressed genes between the OJA and PJA suggest that dynamic developmental gene regulation establishes jaw structure and its major differences early in ontogeny, with later developmental refinement linked to guild-specific trophic adaptation.

### (b) Repeated use of genes in the evolution of herbivory versus carnivory

Trophic diversification is a hallmark of adaptive radiation. While transitions from carnivory to herbivory seem limited to only some fish genera, including cichlids and carps, the repeated evolution of herbivory is rarer but can be observed in modern haplochromines [5]. The small number of shared differentially expressed genes across all species pairs indicates highly guild-specific gene regulatory programmes in LV cichlids, a finding further supported by the variance partitioning analysis. We identified candidate genes that were repeatedly differentially expressed across all species comparisons. The majority of these shared candidate genes (79–96%) between carnivores and herbivores exhibited the same direction of expression. This suggests an important role of these genes in the development of divergent jaw morphologies.

Several of these genes were associated with Wnt signalling, such as the *cmah* gene that was upregulated in carnivores at the larval stage (figure 3A,B). *Cmah* encodes an enzyme that regulates Wnt signalling [31], which plays a crucial role in skeletogenesis of fish jaws (reviewed in [32]). Higher levels of Wnt in Lake Malawi cichlids were also correlated with a more steeply descending head profile, early variation and increased ossification in craniofacial development associated with the evolution of trophic novelty [33]. The Wnt pathway was enriched in the OJA of adults (figure 3F) and was associated with the genes *fam53b* and *bcl9l*. *Fam53b* regulates the Wnt pathway [34], and *bcl9l* has been linked to craniofacial malformations in zebrafish [35]. However, neither of these genes has been previously implicated in cichlid jaw development. In larvae, the GO term ‘roof of mouth development’ (figure 3C) was associated with *sox9b*, which was downregulated in carnivores. *Sox9b* is a transcription factor involved in craniofacial chondrogenesis and shortened jaws in fish [36,37].

Besides genes related to the canonical Wnt/β-catenin signalling pathway, we found *ggt5* to be downregulated in the jaw tissues of larval herbivores. *Ggt5* is a direct downstream target of *runx2*, a major osteogenic transcription factor [38] that is involved in tooth development and adaptation in cichlid fishes [39]. Another gene with reduced expression in both jaws of herbivores and a key role in the stimulation of osteoclastogenesis is *granulin* [40]. Taken together, the expression results for *ggt5a* and *granulin* may imply a distinct bone resorption process between carnivores and herbivores at the larval stage.

In adults, the most consistent expression differences in both jaws (validated by RNAseq and qPCR; supplementary material, figure S5B) between the trophic niches were observed for *kcne4,* showing reduced expression in herbivores. *kcne4* directly interacts with calmodulin [41], which plays an important role in jaw skeletogenesis, contributing to variation in jaw structures among closely related species of Darwin's finch adaptive radiations and cichlid fishes [42–44].

### (c) Alternative splicing contributed to trophic divergence of jaws

We still do not fully understand the role of alternative splicing in adaptive evolution and how it interacts with gene expression regulation [9], the latter of which has been the focus of most evolutionary transcriptomic studies. We understand even less about how alternative splicing changes during development. Our study revealed that, similar to the patterns of differential gene expression, alternative splicing showed tissue-specific patterns at the larval stage, shifting to a guild-specific pattern in adults. Thus, the developmental patterns of both gene expression changes and alternative splicing were mirrored. However, the genes that were differentially expressed and those that were differentially spliced between ecologically divergent species were completely different. A few genes were alternatively spliced in both jaws at both developmental stages in herbivorous and carnivorous species (figure 4). One of the genes that was significantly enriched for the GO biological process term ‘chondrocyte proliferation’ in oral jaws at both developmental stages (larval OJA and adult OJA) was *frzb*, frizzled-related protein. While the two isoforms of this gene showed a guild-specific usage pattern in the larval OJA, a trophic niche-specific pattern in usage emerged in adults (figure 4B,E). Kamel *et al.* [45] showed that *frzb* plays an important role in jaw development and palate morphogenesis, with a knockdown of *frzb* in zebrafish causing the complete loss of the lower jaw. *Frzb* also enhances the diffusion of Wnt by blocking short-range effects and increasing long-range gene responses [46] and plays a role in the convergent extension of the jaw skeleton. This suggests that this might be an underlying molecular basis for the protrusion of the jaw in carnivorous cichlids.

Another gene with different isoform usage between the OJA of larval herbivores and carnivores was *fgfr2*, fibroblast growth factor receptor 2 (figure 4A,D). Mutations in *fgfr2* have been associated with craniosynostosis (the premature fusion of sutures), short hypoplastic maxilla and mandibular prognathism [47,48], potentially linking differences in isoform usage to a steeper jaw profile in herbivores. In cichlids, *fgfr2* is involved in tooth development [49]. Overall, these results suggest that the alternative usage of isoforms shaped the rapid evolution of jaws adapted to different food sources in LV cichlids.

### (d) Exonization generated novel variation for the evolution of herbivory

Exonization, the process of non-coding intronic regions becoming protein-coding exons, can generate new gene isoforms and potentially new proteins [50]. Although most exonization events are thought to be molecular noise, they provide a rapid mechanism for generating new protein-coding genes from the original genome. As long as the original isoform maintains functionality, the new alternative exonized isoform can avoid purging through selection and may evolve a novel function over time [51]. Because exonization can rapidly evolve from standing variation, it has the potential to be an important substrate for adaptive evolution [9]. We found that a transcript of the *kaznb* (Kazrin) gene, which was consistently differentially expressed between all carnivores and herbivores across both developmental stages within each jaw, may have evolved through the exonization of an intron. The *kaznb* gene is known to play an essential role in craniofacial development [52]. *Xenopus laevis* embryos depleted for *kaznb* showed head defects, including smaller ceratohyal and ceratobranchial cartilages and eyes [52]. Exonization may have diversified *kaznb*’s function. Alternatively, the retention of an intron in the reading frame could lead to these isoforms being marked for nonsense-mediated decay—thus decreasing the expression of this gene, which could also have functional downstream impacts [53]. It has been previously shown that herbivorous/carnivorous cichlids from Lake Tanganyika have significantly higher intron retention (i.e. exonization) than omnivorous species [7]. Future studies should validate the function of *kaznb* in the jaws of cichlid fishes through RNA overexpression or CRISPR-Cas9. Exonization may play an underappreciated role in adaptive radiation as a rapid source of protein-coding variation and potential driver of evolutionary novelty.

### (e) Distinct roles of gene expression and alternative splicing during development

A number of studies have empirically investigated the differing roles of differential gene expression and alternative splicing in phenotypic adaptation and found that they play non-redundant roles [7,54,55]. In our study, almost no genes or enriched GO biological processes were both differentially expressed and differentially spliced between species adapted to different trophic niches (supplementary material, figures S8 and S9). Even though these two gene regulatory mechanisms are targeting different genes and pathways, they often involved in the same broader craniofacial developmental process. For example, the alternative splicing GO enrichment showed involvement in ‘chondrocyte proliferation’ (larval OJA, adult OJA and adult PJA), while the differential expression GO enrichment was associated with terms such as ‘roof of mouth development’ (larval OJA) and ‘Wnt signalling pathway’ (adult OJA). Taken together, our results suggest that modulation of gene expression and splicing plays distinct roles in shaping developmental gene regulatory programmes that underlie ecologically important jaw adaptations in cichlid fishes. A meta-analysis of gene expression and alternative splicing across broader taxonomic groups could help shed light on their evolutionary relationship beyond single lineages.

### (f) Limitations and future directions

Our study is one of the first to analyse transcriptome-wide differences in multiple species of East African cichlids across more than one developmental stage. However, there were several methodological decisions that had to be made to enable this analysis that have implications for the interpretation of the results. Firstly, owing to the small size of larvae of LV cichlids at stage 26, dissections included associated tissues, such as muscle and cartilage, that could not be removed even in the adults. This precluded the direct comparison of jaws in adults versus larvae in our analyses. Secondly, owing to the size of the larvae, two individuals had to be pooled to obtain enough RNA for one library preparation. While this may impact the individual-level expression signals, it does not impact the guild-specific expression signals that we are interested in. In the future, both these limitations can be overcome through single-cell RNA sequencing [56]. At present, we cannot disentangle whether expression differences that we found between herbivorous and carnivorous species arose from shared ancestry or de novo. In the future, expanding this dataset to include more species and account for phylogeny may potentially shed light on this. Validating candidate isoforms identified here using *in silico* protein prediction, RT-PCR and gene editing approaches would be valuable next steps.

## 4. Conclusion

Little is known about the developmental dynamics of gene expression and alternative splicing evolving at very short time scales, as most studies have focused on older lineages and at single life stages. Here, we show that both differential expression and differential splicing are important and non-redundant gene regulatory processes underlying rapid morphological change during development. Future studies would benefit from including multiple developmental stages in their study design and broader phylogenetic sampling.

## 5. Material and methods

### (a) Sampling

In this study, we focus on five model species from LV (*Gh*, *Ht*, *Ml*, *No* and *Ps*). We picked those five species to try to reflect the broad spectrum of trophic specializations in LV (details in supplementary material). The OJA samples contained the premaxilla, maxilla, dentary and articular, while the PJA samples were composed of the upper and lower pharyngeal jaws. In terms of larvae, the area of the OJA and PJA was sampled, and two individuals were pooled (sex could not be determined). All adult samples were males (showing nuptial colouration as a sign of adulthood). RNA extraction and library preparation were conducted for all of these samples (details in supplementary material).

### (b) Transcriptome assembly

For each of the samples, we got an average of ten million paired-end reads. Sequence reads are available at the NCBI Sequence Read Archive (SRA) under the accession number PRJNA640176. After a quality check with FastQC (v0.11.8) [57] and a trimming step with Trimmomatic (v0.3.9) [58], only reads with a Phred score > 28 and a minimum length of 70 bp were retained. For each sample, a median of about 5 million reads was obtained. For read alignment, we used STAR (v2.7.3.a) [59] with the *Oreochromis niloticus* genome reference from the University of Maryland [25] (*O. niloticus*; NCBI accession number: GCA_001858045.3) as the guide reference, as it is very well annotated. After checking the mapping statistics with samtools idxstats (v1.9) [60] and merging the single files for the same species and jaw type with Picard (v2.21.7) [61], we used StringTie (v2.0.6) [62] without a reference to assemble the RNA-seq alignments into potential transcripts. To minimize false positives, we implemented a hierarchical merging strategy: transcript assemblies were generated for both individual replicates (per fish) and merged files (per species and jaw type via Picard (v2.21.7) [61]. These were then progressively combined—first by species, then by tissue, and subsequently across all tissues and developmental stages—to produce a final, unified isoform annotation. To estimate the accuracy of the produced annotation files, we compared them with gffcompare (v0.11.2) [63] to our reference annotation. We filtered for monoexonic transcripts not contained in the reference and a class code assigned by gffcompare, indicating ‘possible polymerase run-on’ fragments. The maximum intron length was reduced to 200 000 bp, which is the maximum intron length in the *O.niloticus* reference. Based on these annotations, the expression estimates were generated with StringTie, allowing no multi-mapping. From these expression estimates, count matrices were produced using the prepDE.py script (StringTie v2.0.6) to extract raw count data from StringTie results. The code used for the whole analysis is stored in a GitHub repository.

### (c) Differential gene expression analysis

Adult and larval jaws could not be directly compared because identical dissections were impossible at the larval stage owing to the small body size. We, therefore, avoided direct stage comparisons and instead performed the same species-pairwise contrasts within each life stage. We then examined the intersection sets across contrasts to identify shared gene-expression and alternative-splicing patterns associated with trophic niche. We used DESeq2 [64] in [65] using RStudio [66] to detect differentially expressed genes by running comparisons between all species and tissues, as well as comparisons between the tissues within a species (supplementary material). This was done by creating a combined group factor encoding, including tissue and species identity (e.g. ‘OJGh’ and ‘PJMl’) and using that as the single design variable. This parametrization allows all between-species and between-tissue contrasts to be extracted from a single fitted model, which shares dispersion estimates across all groups. The full set of between-species contrasts comprised 10 per tissue-stage combination (all pairwise combinations of five species), plus five within-species OJA versus PJA contrasts per stage (supplementary analysis), for a total of 25 contrasts per stage (50 across both stages). DESeq2 uses raw read counts and estimates variance-mean dependence based on a model that uses a negative binomial distribution [64]. Significance was assessed using the DESeq2 adjusted *p*-value (padj < 0.05). To further strengthen the validity of our approach, we also included a signed Stouffer analysis (FDR < 0.05) with full direction consistency using the *p*-values and log2 fold-changes of the differential gene expression analysis of the herbivore-carnivore comparisons to cross-validate our intersection sets.

Attempting to separate guild-identity variation from trophic niche signal, we also ran a variance partitioning analysis. For the variance partitioning, we used the raw gene count matrices as input. Counts were first filtered, TMM-normalized with edgeR [67] and converted to log-CPM values. Variance partitioning was then applied to these TMM-normalized log-CPM values. The main model was: approximately (1|diet) + (1|species) + (1|experimental) + (1|sample), where experimental stands for the oral and pharyngeal jaw apparatus.

### (d) Gene Ontology term enrichment

GO terms for *O. niloticus* were acquired in R [65] using RStudio [66] via the biomaRt package (v2.46.1) [68,69] in R from the Ensembl database. Gene set enrichment analysis was conducted with the R program topGO (v2.36.0) [70] using the *weight* method to account for GO topology and Fisher's exact test for the enrichment analysis.

### (e) Alternative splicing analysis

We applied differential transcript usage analysis to investigate differences in transcript usage between the different species and trophic niches. Differential transcript usage was analysed with SUPPA2 (v2.3) [26] using StringTie transcript-level TPM values. SUPPA2 takes the normalized isoform counts of all replicates of a condition and calculates a relative abundance for each transcript in each sample (PSI). To detect the difference in transcript usage between conditions, dPSI is computed (dPSI = PSI condition two − PSI condition one) [26]. Herbivore-carnivore species contrasts were tested with SUPPA2 diffSplice using empirical bootstrap *p*-values (-m empirical) and expression-level correction (-gc). Events/transcripts with >33% missing PSI values were removed (-nan 0.33). Differentially used transcripts were called at *p* < 0.05. Median PSI across five biological replicates per species was used for visualization in R [65] using RStudio [66].

## Supplementary Material



## Data Availability

Sequence reads are available at NCBI sequence read archive (SRA) under the accession number PRJNA640176 (https://www.ncbi.nlm.nih.gov/sra/?term=PRJNA640176). The code for the analysis is available on Zenodo [71]. Supplementary material is available online [72].
