# Supplementary material for: Developmental divergence in gene regulation underlies trophic innovation in Lake Victoria cichlids
Source: Proc Biol Sci. 2026 Oct 7;293(2080):20253318. doi: 10.1098/rspb.2025.3318 (PMC13641903; doi:10.1098/rspb.2025.3318)

Developmental divergence in gene regulation underlies trophic innovation in Lake Victoria cichlids

Anna Duenser*, Pooja Singh*, Ehsan Pashay Ahi, Marija Durdevic, Sylvia Schaeffer, Julian Gallaun,Wolfgang Gessl, Ole Seehausen, Christian Sturmbauer

*These authors contributed equally

Correspondence: Anna Duenser (anna.duenser@gmail.com), Pooja Singh (pooja.singh09@gmail.com)

doi: 10.1098/rspb.2025.3318; published in: Proceedings of the Royal Society B

Supplementary data:

Supplementary Methods: Sampling, RNA extraction, Library preparation and sequencing, qPCR analysis, Quantitative PCR (qPCR) confirming gene expression.

Supplementary Figure S1: *Scans of pharyngeal jaw apparatus (PJA) of selected species. Larval PJA of H. thereuterion (A), adult PJA of H. thereuterion (male) (B), larval PJA of N. omnicaeruleus (C) and adult PJA of N. omnicaeruleus (male) (D), larval PJA of P. sauvagei (E), adult PJA of P.sauvagei (male) (F). H. thereuterion (dark orange; carnivore), N. omnicaeruleus (dark blue; herbivore), P. sauvagei (pink; omnivore).*

Supplementary Figure S2: *Transcriptome-wide results across two developmental stages were investigated for each species: larvae (developmental stage 26 as defined by Fujimura and Okada (*Fujimura and Okada 2007*) and adults (onset of sexual maturity). Normalised gene expression PCA plot for larvae (E) and adults (G) based on 59,849 normalised isoform expression counts. (F) and (H) show dendrograms of the Euclidean distance based on 59,849 normalised isoform expression counts in larvae (F) and adults (H). Dendrogram annotations: Oral jaw apparatus (OJA; black), pharyngeal jaw apparatus (PJA; white). PCA annotations: OJA are circles and PJA are triangles. Dendrogram and PCA annotations: G. hiatus (Gh; bright orange; carnivore), H. thereuterion (Ht; dark orange; carnivore), M. lutea (Ml; bright blue; herbivore), N. omnicaeruleus (No; dark blue; herbivore) and P. sauvagei (Ps; pink; omnivore).*

Supplementary Figure S3: *PCA plot for larvae (A) and adults (C) based on normalised expression of 37,334 genes including the outgroup species Tropheops tropheops (Tt) and Astatotilapia burtoni (Ab). (B) and (D) show dendrograms of the Euclidean distance for larvae (B) and (D) adults. Dendrogram annotations: Oral jaw apparatus (OJA; black), pharyngeal jaw apparatus (PJA; white). PCA annotations: OJA are circles and PJA are triangles. Dendrogram and PCA annotations: A. burtoni (Ab; grey; omnivore),G. hiatus (Gh; bright orange; carnivore), H. thereuterion (Ht; dark orange; carnivore), M. lutea (Ml; bright blue; herbivore), N. omnicaeruleus (No; dark blue; herbivore) and P. sauvagei (Ps; pink; omnivore), T. tropheops (Tt; black, herbivore).*

Supplementary Figure S4: *Heatmaps of the normalised counts of differentially expressed genes from comparisons including carnivorous versus herbivorous species for the larval oral (A) and pharyngeal jaw apparatuses (B) as well as the adult oral (C) and pharyngeal jaw apparatuses (D). Annotated genes are indicated for each heatmap. Oral jaw apparatus (OJA), pharyngeal jaw apparatus (PJA), G. hiatus (Gh; bright orange; carnivore), H. thereuterion (Ht; dark orange; carnivore), M. lutea (Ml; bright blue; herbivore), N. omnicaeruleus (No; dark blue; herbivore) and P. sauvagei (Ps; pink; omnivore).*

Supplementary Figure S5: *Relative mRNA expression analysis results for selected genes via qPCR for larval oral and pharyngeal jaw (A) and adult oral and pharyngeal jaw apparatus(B). Bars*

show the standard deviation for RQ values of three biological replicates per jaw tissue. Colored circles refer to significance between comparisons ($p < 0.05$). Scale differs for each gene. Oral jaw apparatus (OJA), pharyngeal jaw apparatus (PJA), G. hiatus (Gh; bright orange; carnivore), H. thereuterion (Ht; dark orange; carnivore), M. lutea (Ml; bright blue; herbivore), N. omnicaeruleus (No; dark blue; herbivore).

Supplementary Figure S6: *Upset plots of overlapping differentially used transcripts (DTU) for species comparisons including the carnivorous and herbivorous species. Yellow and green brackets highlight carnivorous and herbivorous species, respectively. Points below bars show overlapping transcripts between indicated pairwise comparisons or unique sets of genes for each comparison. Light grey bars indicate the number of overlapping genes between all pairwise species comparisons. Upset plots are shown for larval OJA (A), larval PJA (B), adult OJA (C) and adult PJA (D). Venn diagrams show the overlap in larval and adult differentially used transcripts for OJA (E) and PJA (F). Oral jaw apparatus (OJA), pharyngeal jaw apparatus (PJA), G. hiatus (Gh), H. thereuterion (Ht), M. lutea (Ml) and N. omnicaeruleus (No). Oral jaw apparatus: OJA, pharyngeal jaw apparatus: PJA, G. hiatus (Gh; bright orange; carnivore), H. thereuterion (Ht; dark orange; carnivore), M. lutea (Ml; bright blue; herbivore), N. omnicaeruleus (No; dark blue; herbivore).*

Supplementary Figure S7: *Stacked bar plots of isoforms with differential usage between at least one carnivore-herbivore comparison. Median PSI values (relative abundance) were calculated across 5 replicates each. Different shadings imply different isoforms: green (isoform 1), light grey (isoform 2), black (isoform 3), dark grey (isoform 4) and yellow (isoform 5). Barplots are shown for larval OJA (A), larval PJA (B), adult OJA (C) and adult PJA (D). Oral jaw apparatus (OJA), pharyngeal jaw apparatus (PJA), G. hiatus (Gh; carnivore), H. thereuterion (Ht; carnivore), M. lutea (Ml; herbivore) and N. omnicaeruleus (No; herbivore).*

Supplementary Figure S8: *Overlap of differentially expressed genes (DEG) and alternatively spliced genes (AS) at the larvael (L) or adult (A) stage in the oral jaw apparatus (OJA) and pharyngeal jaw apparatus (PJA) between herbivores and carnivores.*

Supplementary Figure S9: *Overlap of Gene Ontology (GO) enrichment terms in the genes that were alternatively spliced (AS) or differentially expressed (DEG) at the larvael (L) or adult (A) stage in the oral (OJA) and pharyngeal jaw apparatus (PJA).*

Supplementary analysis:
Gene expression modularity of the two jaws, Candidate genes for cichlid jaw modularity

Supplementary Figure S10: *Bar plots show the number of differentially expressed genes between oral and pharyngeal jaw apparatus for each species in the larval (A) and adult (B) stage. The indicated line shows the number of genes that are shared across all species for each stage. Heatmaps show the gene expression pattern of 121 overlapping genes between the larval (C) and adult (D). Oral jaw apparatus (OJA), pharyngeal jaw apparatus (PJA), G. hiatus (Gh), H. thereuterion (Ht), M. lutea (Ml), N. omnicaeruleus (No) and P. sauvagei (Ps).*

Supplementary Figure S11: *The Venn diagram shows the overlap in larval and adult differentially expressed genes in the oral versus the pharyngeal jaw apparatus and their direction of expression (upregulated genes are indicated by the upward facing arrow in green, downregulated genes by the downward facing arrow in red; direction indicated for OJA versus PJA) (A). Selected GO terms are listed in (B).*

Supplementary Figure S12 & 13: *Normalised gene expression PCA plot for larvae and adult samples based on 37,334 genes to confirm that no batch effect was introduced via library prep/ sequencing.*

Volcano plots of all differential gene expression analyses of all species-species comparisons: Pages 24-44 of Supplemental Material. *G. hiatus (Gh), H. thereuterion (Ht), M. lutea (Ml), N. omnicaeruleus (No), P. sauvagei (Ps), oral jaw apparatus (OJ), pharyngeal jaw apparatus (PJ), adult (A), larvae (L).*

Supplementary Tables:

Table S1: *Number of differentially expressed genes for all species-species comparisons. G. hiatus (Gh), H. thereuterion (Ht), M. lutea (Ml), N. omnicaeruleus (No), P. sauvagei (Ps), oral jaw apparatus (OJA), pharyngeal jaw apparatus (PJA).*

Table S2: *Percentage of genes and shared direction of expression. Oral jaw apparatus (OJA), pharyngeal jaw apparatus (PJA).*

Table S3: *Number of differentially used isoforms for herbivorous versus carnivorous comparisons. G. hiatus (Gh), H. thereuterion (Ht), M. lutea (Ml), N. omnicaeruleus (No), P. sauvagei (Ps), oral jaw apparatus (OJA), pharyngeal jaw apparatus (PJA).*

Supplementary Files:

Supplementary File 1: *Raw and trimmed read counts, mapping statistics and accession numbers of all samples.*

Supplementary file 2: *GO ids for shared differentially expressed genes in larval and adult oral and pharyngeal jaw apparatus. Larvae (L); adult (A); differentially expressed genes (DEG); oral jaw apparatus (OJA); pharyngeal jaw apparatus (PJA).*

Supplementary File 3: *Shared differentially expressed genes (FDR ≤ 0.05) between herbivorous and carnivorous species including the direction of expression (log2FC_dir). Larvae (L); adult (A); differentially expressed genes (DEG); oral jaw apparatus (OJA); pharyngeal jaw apparatus (PJA).*

Supplementary file 4: *Shared differentially spliced isoforms between herbivorous and carnivorous species in larval and adult oral and pharyngeal jaw apparatus. Larvae (L); adult (A); oral jaw apparatus (OJA); pharyngeal jaw apparatus (PJA).*

Supplementary file 5: *GO ids for shared differentially spliced isoforms in larval and adult oral and pharyngeal jaw apparatus. Larvae (L); adult (A); alternative splicing (AS); oral jaw apparatus (OJA); pharyngeal jaw apparatus (PJA).*

Supplementary Movies:

H_thereuterion_adult_PJA.mp4: Pharyngeal jaw apparatus (PJA) of an adult *H. thereuterion*.

H_thereuterion_stage26_PJA.mp4: Pharyngeal jaw apparatus (PJA) of *H. thereuterion* at stage-26.

N_omnicaeruleus_adult_PJA.mp4: Pharyngeal jaw apparatus (PJA) of an adult *N. omnicaeruleus*.

N_omnicaeruleus_stage26_PJA.mp4: Pharyngeal jaw apparatus (PJA) of *N. omnicaeruleus* at stage-26.

P_sauvagei_adult_PJA.mp4: Pharyngeal jaw apparatus (PJA) of an adult *P. sauvagei.*

P_sauvagei_stage26_PJA.mp4: Pharyngeal jaw apparatus (PJA) of *P. sauvagei* at stage-26.

Supplementary Methods:

Sampling

G. hiatus feeds on chironomid larvae as well as on bivalves and mollusks (Witte et al. 1982). *H. thereuterion* mostly feeds on insects like chironomid larvae, but a substantial portion of its nutrition is covered by collecting terrestrial insects from the water surface (Oijen et al. 1996). They both represent differentially specialised carnivores. *M. lutea* and *N. omnicaeruleus* are both mainly herbivorous but with different modes of feeding. While *M. lutea* feeds mainly on filamentous algae and also takes in some detritus, *N. omnicaeruleus* grazes off epilithic algae and diatoms (Seehausen et al. 1998). *P. sauvagei* is an omnivore, feeding on epilithic algae, associated insects and sponges (Seehausen 1996). As an outgroup we included *Tropheops tropheops* (Tt), an herbivorous Lake Malawi cichlid, which mainly feeds on algae and scratches it from hard surfaces, but has also been reported to exclusively feed on plankton if abundant (Konings 1990). As a second outgroup species we selected *Astatotilapia burtoni* (Ab), a mostly riverine cichlid that is located in Lake Tanganyika and its surrounding waterways and can be classified as an omnivore, feeding on plants, aufwuchs, insects and fish remains (Muschick et al. 2012). We scanned the pharyngeal jaw apparatus of *H. thereuterion*, *N. omnicaeruleus* and *P. sauvagei* at the larval and the adult stage to visualise the differences in morphology that are visible from the beginning of the exogenous feeding (Fig. S9). Scans were conducted with a Scanco Medical µCT 40 and images were created with the 3D Slicer version 5.6.2.

The *M. lutea* were collected from Makobe Island and the *G. hiatus* from the northern Mwanza Gulf. Both were collected by Ole Seehausen. The other samples, *N. omnicaeruleus*, *P. sauvagei* and *H. thereuterion* were obtained via aquarium trade.

All fish were reared in the fish facility at University of Graz under standardised aquarium conditions regarding tank environment, diet and water parameters. Eggs were taken from the mouth of their mother 24 hours after fertilisation, by applying slight pressure onto their cheeks, and reared in a separate tank until the yolk sac was completely absorbed (stage 26, (Fujimura and Okada 2007)). The larvae were euthanized with an overdose of MS 222 and stored in RNAlater in a fridge for seven days and then moved to a -80°C freezer. Fish raised to the adult life stage were fed with a standardised diet of green spirulina flakes. When individuals started to show

adult colouring and breeding behavior males were sacrificed with MS 222 and dissected. Bones were stored in RNAlater in a fridge for seven days and then frozen at -80°C.

Dissection and RNA extraction

Per species and stage five biological replicates of oral and pharyngeal jaw apparatus samples were taken. The oral jaw apparatus samples contained the premaxilla, maxilla, dentary and articulate while the pharyngeal jaw apparatus samples were composed of the upper and lower pharyngeal jaw. In the case of the larvae the area of the OJA and of the PJA were sampled under a stereo microscope and submerged in a lysis buffer and 1-thioglycerol according to the protocol of the Promega RNA Reliaprep kit. This kit includes filter columns and a DNA digestion step. The sample in the lysis buffer was crushed with a ceramic bullet in a homogenizer (FastPrep-24, MP Biomedicals, Santa Ana, CA, USA), before further processing. Two individuals were pooled per sample to increase the amount of RNA. For the adult species the bones were all cleaned from skin and muscle tissue and stored in RNAlater, this was not possible for the larvae. As the whole set of bones would have been too much tissue input for the RNA extraction, only half of the bones were collected. The set of the bones is the same for all samples, but if bones for RNA extraction were collected from the left or right side was randomly decided by a simple randomization script in R (R Core Team 2020) prior to each extraction. RNA for the adults was extracted using TRIzol reagent (Thermo Fisher Scientific), following the protocol of the TRIzol protocol for RNA extraction with the inclusion of a DNAse step at the end (invitrogen). RNA quality was tested at a TapeStation 2200 with RNA Screen Tapes. It was tried to reach a RINe number of >= 7 for each sample.

Library preparation and sequencing

Library preparation was done with the TruSeq Stranded mRNA Sample Prep Kit using 24 indexing adapters and following the standard protocol. For the input RNA we tried to reach 1000 ng for each sample. The quality of the cDNA was tested on a TapeStation 2200 (Agilent) with D1000 Screen Tapes from the Agilent RK6 ScreenTape System. Sequencing was executed at the Vienna BioCenter Core

Facilities on a HiSeq 2500 with paired end 125 cycles per read (2 x 125 cycles). Demultiplexing was conducted by the same facility.

qPCR analysis

From four replicates of RNA samples of each jaw apparatus, stage and species, we used 500 ng of RNA to synthesise first strand cDNA using the High Capacity cDNA Reverse Transcription kit (Applied Biosystems). The cDNA from each sample was diluted 1:10 times in nuclease-free water to be used for qPCR. For the adult stage, we selected five candidate target genes; *kcne4*, *kaznb*, *mcl1b*, *rnf114* and *slc5a8*, and for the larval stage we also chose five genes; *ggt5a*, *kaznb*, *granulin*, *faah* and *cmah* (Fig. S5). We used *rpl18* as a reference gene, which we have already validated as a stably expressed gene in both cichlid jaw tissues across different trophic niches (Ahi et al. 2019), to normalise our expression data for qPCR. The primers were designed at conserved sequence regions of the coding sequences from all species using CLC Genomic Workbench, version 7.5 (CLC Bio, Aarhus, Denmark) for alignment and delineation of exon boundaries using *A. burtoni* annotated genome in the Ensembl database (<http://www.ensembl.org>) (Zerbino et al. 2018). Primer Express 3.0 (Applied Biosystems, CA, USA) and OligoAnalyzer 3.1 (Integrated DNA Technology) were used to design the primers as described in details in our previous studies (Ahi et al. 2018; Ahi et al. 2019) (Supplementary data for primer info). Maxima SYBR Green/ROX qPCR Master Mix (2X) (Thermo Fisher Scientific, Germany) was used to generate qPCR reactions. The amplification steps were conducted in 96 well-PCR plates on ABI 7500 real-time PCR System (Applied Biosystems). The primer efficiency calculation, qPCR program, plate set-up and a dissociation step were performed as described previously (Ahi et al. 2018; Ahi et al. 2019). To normalise the expression levels, the Cq value of *rpl18* was used as normalisation factor (Cq reference), and Cq of each target gene was calculated accordingly (ΔCq target = Cq target - Cq reference). Relative expression quantities (RQ) were calculated for the normalised values using E-ΔCq (Pfaffl 2001) and then fold difference values were calculated by transformation of RQ values to logarithmic values in order to conduct further statistical analysis (Bergkvist et al. 2010). The statistical expression differences were identified using ANOVA statistical tests, followed by Tukey’s HSD post hoc tests.

Quantitative PCR (qPCR) confirming gene expression

To validate our findings concerning differentially expressed genes between carnivores and herbivores we conducted qPCR analysis for five candidate genes for trophic divergence at the stage 26 larvae and four candidate genes for trophic divergence at the adult stage, which were found to be differentially expressed in both jaw apparatuses in our differential gene expression analysis. For the larval stage these included *cmah*, *ggt5a*, *granulin*, *kaznb* and *mpc1* and for the adult stage *kaznb*, *kcne4*, *mcl1b*, *slc5a8*. All of these differentially expressed genes showed the same direction of expression between the carnivores and the herbivores in our differential gene expression analysis. While not all of the qPCR differences were significant, they followed similar trends as our differential gene expression analysis results (Fig. S4). *cmah* was consistently upregulated in the differential gene expression analysis in both tissues for all carnivorous species (*G. hiatus and H. thereuterion*) at the larval stage (Fig. 4 A; Fig. S4 A), while the qPCR analysis for *cmah* in both larval tissues showed a significant difference in expression only between *N. omnicaeruleus* and *H. thereuterion* in the OJA and both herbivores (*M. lutea and N. omnicaeruleus*) compared to *H. thereuterion* in the PJA (Fig. 4 B). The differential expression analysis of *mpc1* showed an upregulation in larval herbivores (*M. lutea and N. omnicaeruleus*) in the PJA, which can be seen in *N. omnicaeruleus* in the qPCR analysis, but the difference is not significant (Fig. S4 A). The gene expression of *kaznb* was upregulated in herbivores versus carnivores in all tissues and all stages in the differential gene expression analysis. The qPCR analysis confirmed this pattern in both larval tissues, but at the adult stage, relative mRNA expression in the OJA of *kaznb* could not be detected, while in the PJA, a significant difference was detected only between *H. thereuterion* and *N. omnicaeruleus* (Fig. 4 E). The gene *slc5a8* did also not show significant differences between carnivorous and herbivorous species in relative mRNA expression in the adult OJA, despite it being upregulated in herbivorous species in the differential gene expression analysis (Fig. S4 B).

References

Ahi EP, Singh P, Duenser A, Gessl W, Sturmbauer C. 2019 Divergence in larval jaw gene expression reflects differential trophic adaptation in haplochromine cichlids prior to foraging. *BMC Evol. Biol*. **19**(1), 1–14. (doi: 10.1186/s12862-019-1483-3)

Ahi EP, Singh P, Lecaudey LA, Gessl W, Sturmbauer C. 2018 Maternal mRNA input of growth and stress-response-related genes in cichlids in relation to egg size and trophic specialization. *EvoDevo*. **9**(1):1–17. (doi: 10.1186/s13227-018-0112-3)

Bergkvist A, Rusnakova V, Sindelka R, Garda JMA, Sjögreen B, Lindh D, Forootan A, Kubista M. 2010 Gene expression profiling - Clusters of possibilities. *Methods*. **50**(4), 323–335. (doi: 10.1016/j.ymeth.2010.01.009)

Konings A. 1990 Konings's book of cichlids and all the other fishes of lake malawi. T.F.H. Publications.

Muschick M, Indermaur A, Salzburger W. 2012 Convergent Evolution within an Adaptive Radiation of Cichlid Fishes. *Curr. Biol*. **22**(24),2362–2368. (doi: 10.1016/j.cub.2012.10.048)

Oijen MJP van, Witte F. 1996 Taxonomical and ecological description of a species complex of zooplanktivorous and insectivorous cichlids from Lake Victoria. *Zoologische Verhandelingen*. **302**(1):1- 56.

Pfaffl MW. 2001 A new mathematical model for relative quantification in real-time RT-PCR. *Nucleic Acids Res*. **29**(9), 45e–45. (doi: 10.1093/nar/29.9.e45)

R Core Team. 2020 R: A language and environment for statistical computing. Vienna, Austria: R Foundation for Statistical Computing. See: https://www.R-project.org/

Seehausen O. 1996 Lake Victoria rock cichlids: taxonomy, ecology, and distribution. Verduyn Cichlids, Germany.

Seehausen O, Lippitsch E, Bouton N, Zwennes H. 1998. Mbipi, the rock-dwelling cichlids of Lake Victoria: description of three new genera and fifteen new species (Teleostei). *Ichthyol. Explor. of Fres*. 9(2):129–228.

Witte F, Barel CDN, Hoogerhoud RJC. 1982 The Ecological Differentiation of Two Closely Resembling Haplochromis Species From Lake Victoria (H. Iris and H. Hiatus ; Pisces, Cichlidae). *Neth. J. Zool.* **33**(3), 283–305. (doi: 10.1163/002829683X00147)

Zerbino DR, Achuthan P, Akanni W, Amode MR, Barrell D, Bhai J, Billis K, Cummins C, Gall A, Girón CG, et al. 2018 Ensembl 2018 *Nucleic Acids Res*. **46**(D1), D754–D761. (doi: 10.1093/nar/gkx1098)

Supplementary Figures:

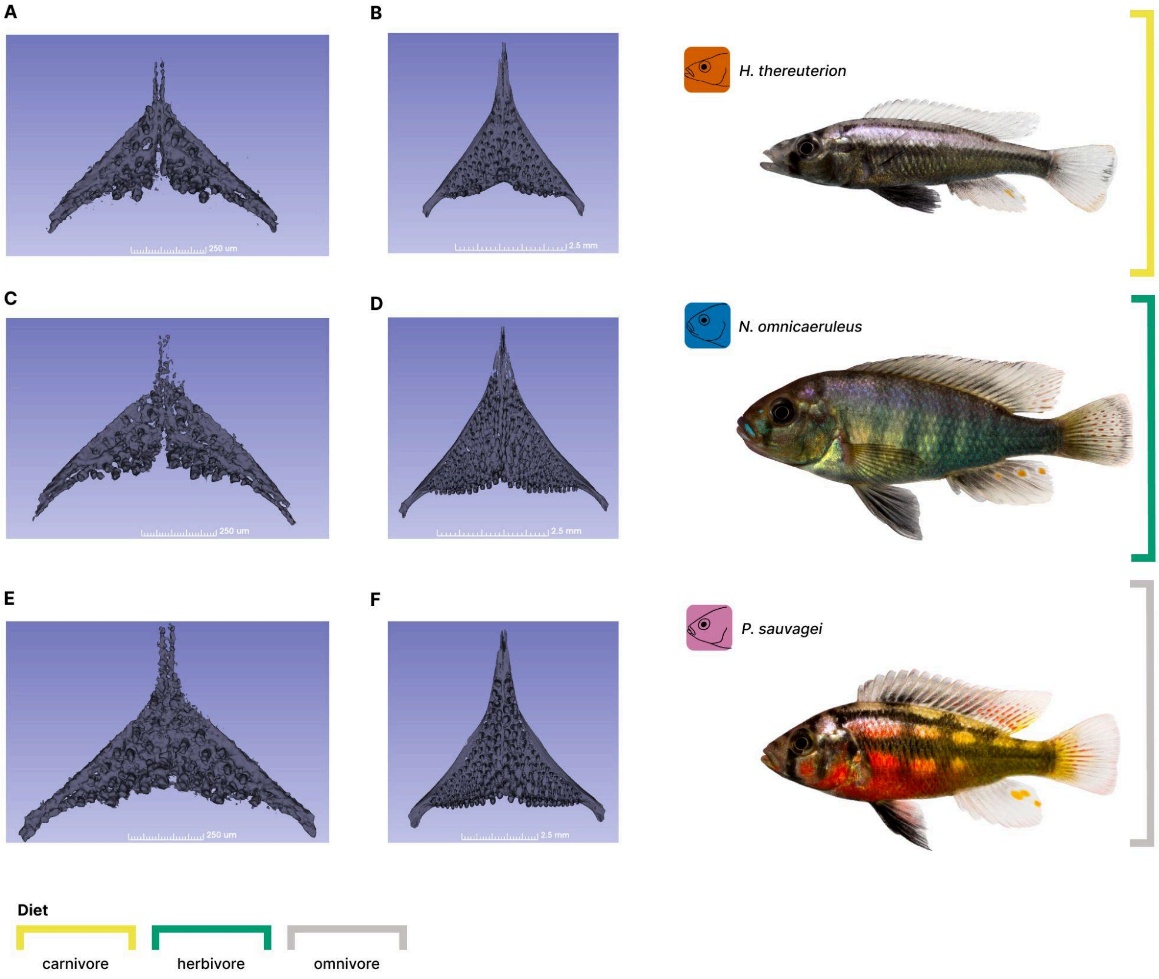

Fig. S1. *Scans of pharyngeal jaw apparatus (PJA) of selected species. Larval PJA of H. thereuterion (A), adult PJA of H. thereuterion (male) (B), larval PJA of N. omnicaeruleus (C) and adult PJA of N. omnicaeruleus (male) (D), larval PJA of P. sauvagei (E), adult PJA of P.sauvagei (male) (F). H. thereuterion (dark orange; carnivore), N. omnicaeruleus (dark blue; herbivore), P. sauvagei (pink; omnivore).*

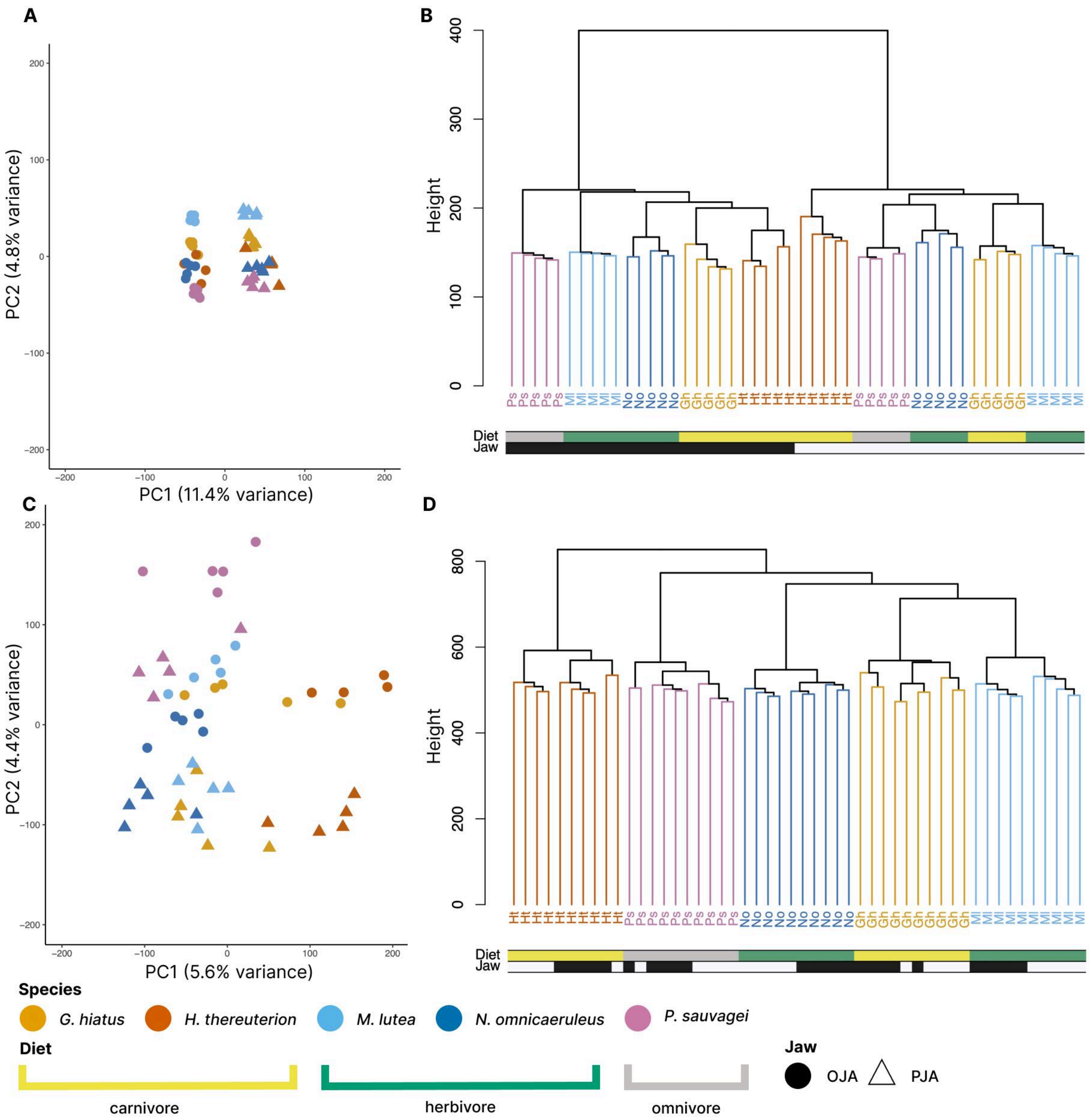

Figure S2: *Transcriptome-wide isoform expression results across two developmental stages were investigated for each species: larvae (developmental stage 26 as defined by Fujimura and Okada (*Fujimura and Okada 2007*) and adults (onset of sexual maturity). Normalised isoform expression PCA plot for larvae (E) and adults (G) based on 59,849 normalised isoform expression counts. (F) and (H) show dendrograms of the Euclidean distance based on 59,849 normalised isoform expression counts in larvae (F) and adults (H). Dendrogram annotations: Oral jaw apparatus (OJA; black), pharyngeal jaw apparatus (PJA; white). PCA annotations: OJA are circles and PJA are triangles. Dendrogram and PCA annotations: G. hiatus (Gh; bright orange; carnivore), H. thereuterion (Ht; dark orange; carnivore), M. lutea (Ml; bright blue; herbivore), N. omnicaeruleus (No; dark blue; herbivore) and P. sauvagei (Ps; pink; omnivore).*

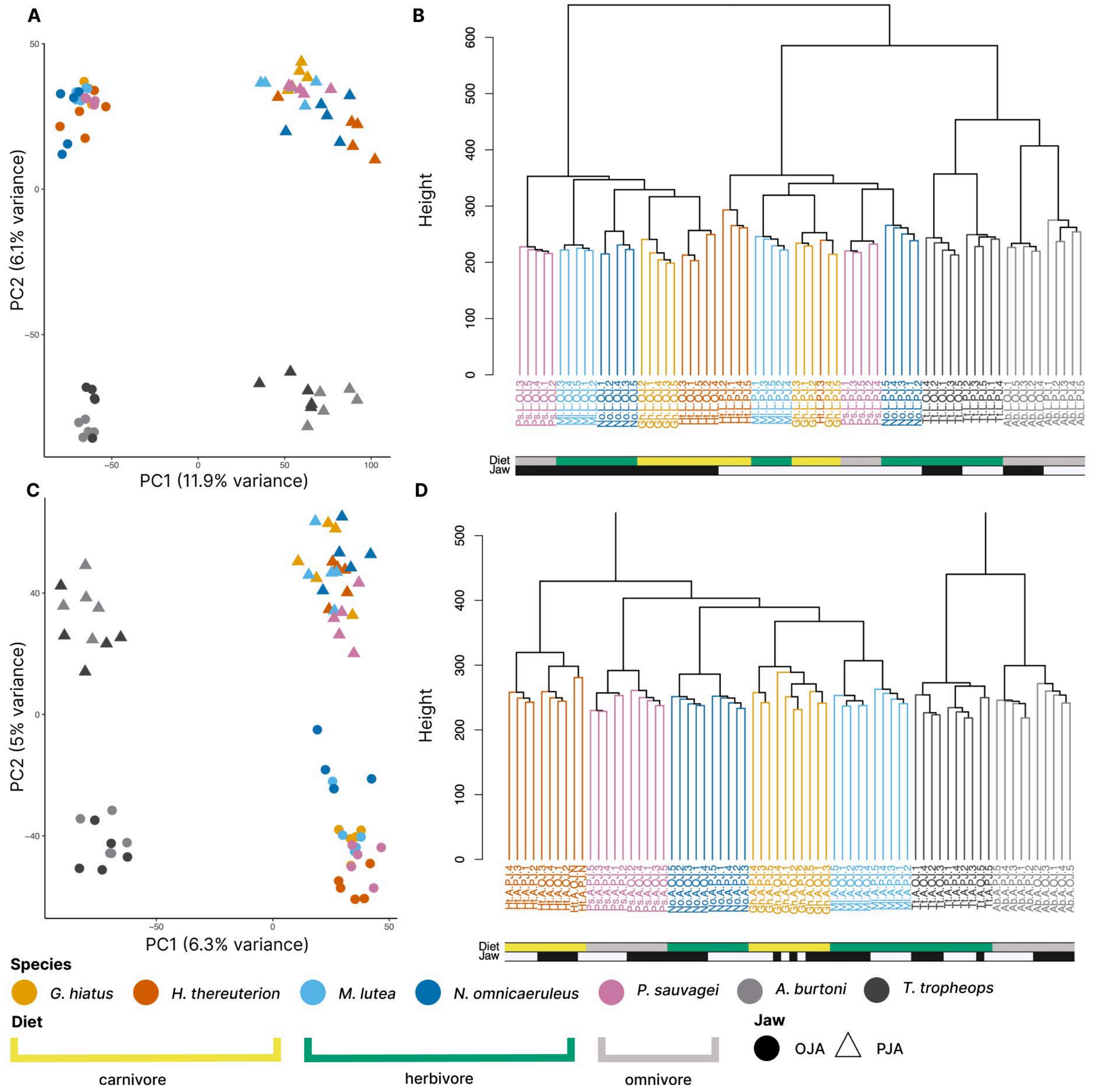

Fig. S3. *PCA plot for larvae (A) and adults (C) based on normalised gene expression of 37,334 genes including the outgroup species Tropheops tropheops (Tt) and Astatotilapia burtoni (Ab). (B) and (D) show dendrograms of the Euclidean distance for larvae (B) and (D) adults. Dendrogram annotations: Oral jaw apparatus (OJA; black), pharyngeal jaw apparatus (PJA; white). PCA annotations: OJA are circles and PJA are triangles. Dendrogram and PCA annotations: A. burtoni (Ab; grey; omnivore),G. hiatus (Gh; bright orange; carnivore), H. thereuterion (Ht; dark orange; carnivore), M. lutea (Ml; bright blue; herbivore), N. omnicaeruleus (No; dark blue; herbivore) and P. sauvagei (Ps; pink; omnivore), T. tropheops (Tt; black, herbivore).*

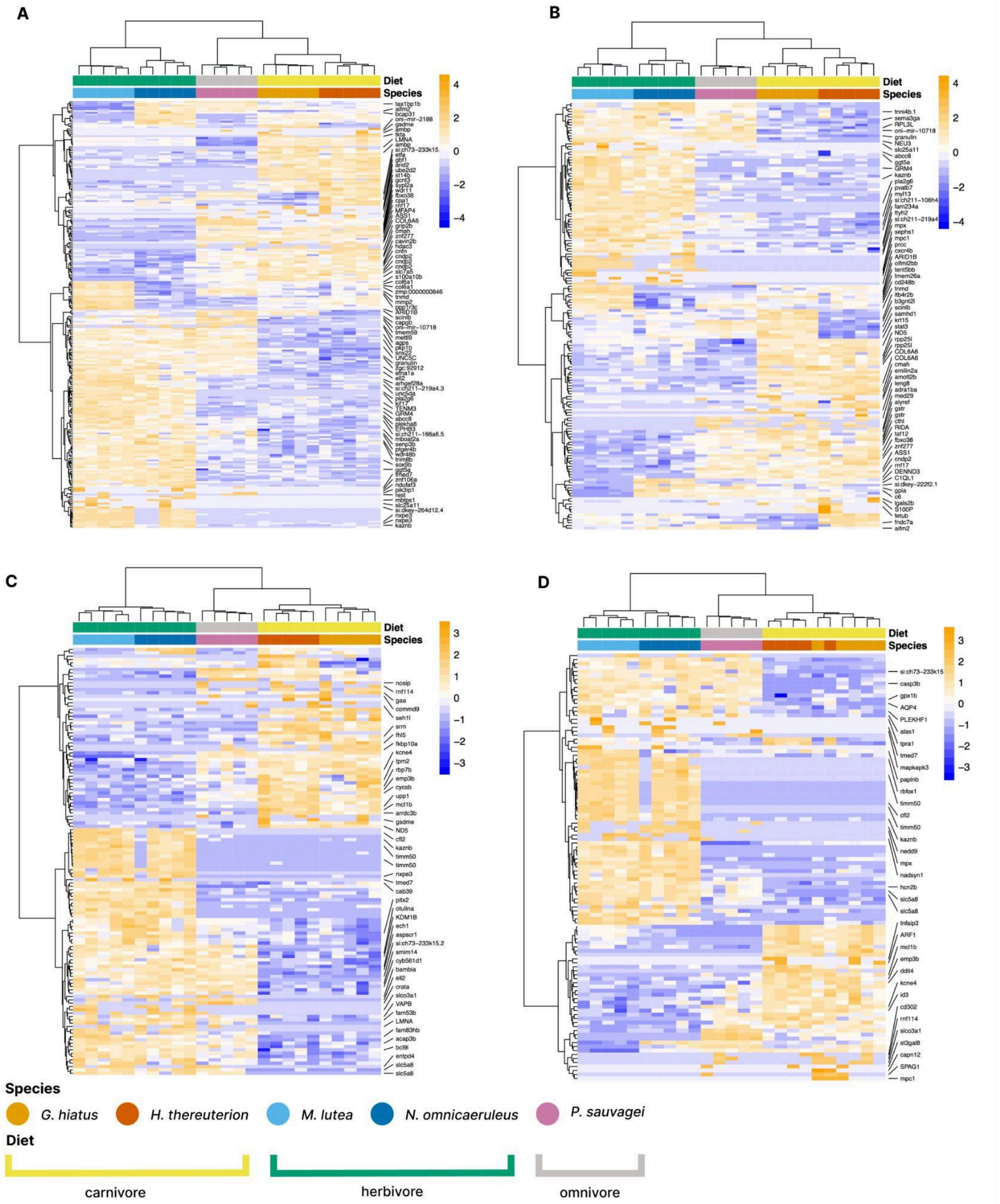

Fig. S4. *Heatmaps of the normalised counts of differentially expressed genes from comparisons between carnivorous versus herbivorous species for the larval oral (A) and pharyngeal (B) jaw apparatuses as well as the adult oral (C) and pharyngeal (D) jaw apparatuses. Annotated genes are indicated for each heatmap. Oral jaw apparatus (OJA), pharyngeal jaw apparatus (PJA), G. hiatus (Gh; bright orange; carnivore), H. thereuterion (Ht; dark orange; carnivore), M. lutea (Ml; bright blue; herbivore), N. omnicaeruleus (No; dark blue; herbivore) and P. sauvagei (Ps; pink; omnivore).*

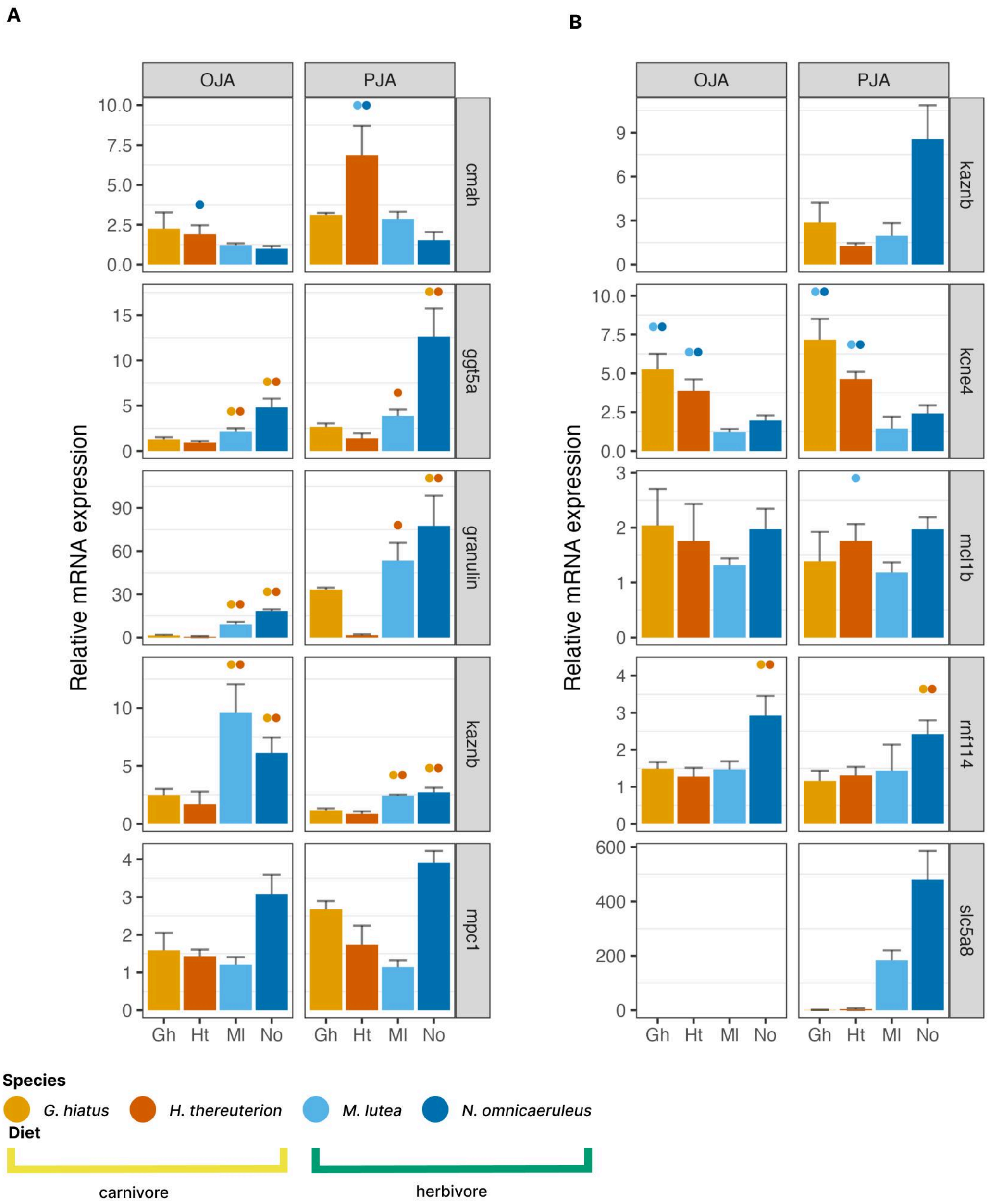

Fig. S5. *Relative mRNA expression analysis results for selected genes via qPCR for larval oral and pharyngeal jaw (A) and adult oral and pharyngeal jaw apparatus(B). Bars show the standard deviation for RQ values of three biological replicates per jaw tissue. Colored circles refer to significance between comparisons (p < 0.05). Scale differs for each gene. Oral jaw apparatus (OJA), pharyngeal jaw apparatus (PJA), G. hiatus (Gh; bright orange; carnivore), H. thereuterion (Ht; dark orange; carnivore), M. lutea (Ml; bright blue; herbivore), N. omnicaeruleus (No; dark blue; herbivore).*

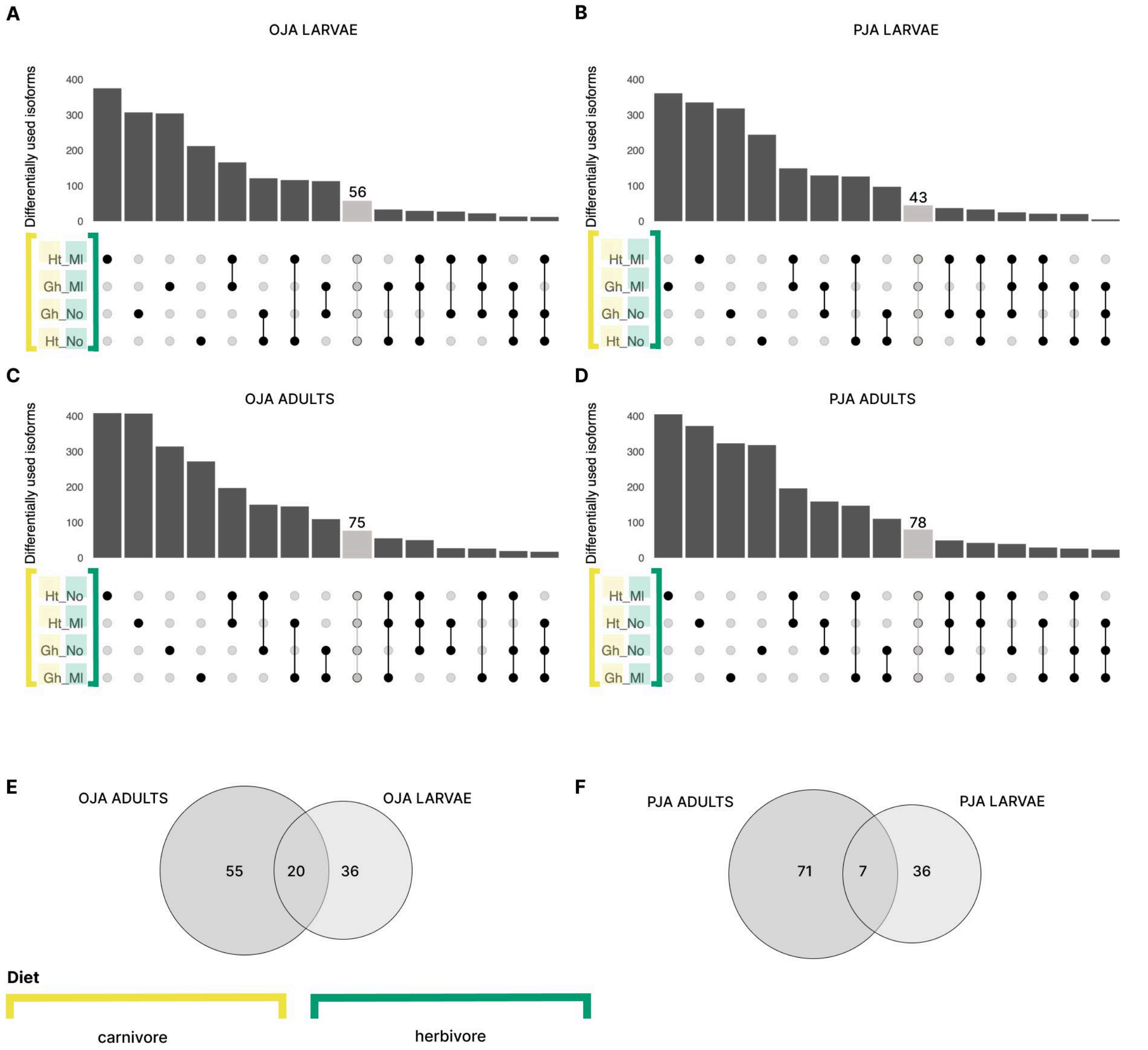

Fig. S6. *Upset plots of overlapping differentially used transcripts (DTU) for species comparisons including the carnivorous and herbivorous species. Yellow and green brackets highlight carnivorous and herbivorous species, respectively. Points below bars show overlapping transcripts between indicated pairwise comparisons or unique sets of genes for each comparison. Light grey bars indicate the number of overlapping genes between all pairwise species comparisons. Upset plots are shown for larval OJA (A), larval PJA (B), adult OJA (C) and adult PJA (D). Venn diagrams show the overlap in larval and adult differentially used transcripts for OJA (E) and PJA (F). Oral jaw apparatus (OJA), pharyngeal jaw apparatus (PJA), G. hiatus (Gh), H. thereuterion (Ht), M. lutea (Ml) and N. omnicaeruleus (No). Oral jaw apparatus: OJA, pharyngeal jaw apparatus: PJA, G. hiatus (Gh; bright orange; carnivore), H. thereuterion (Ht; dark orange; carnivore), M. lutea (Ml; bright blue; herbivore), N. omnicaeruleus (No; dark blue; herbivore).*

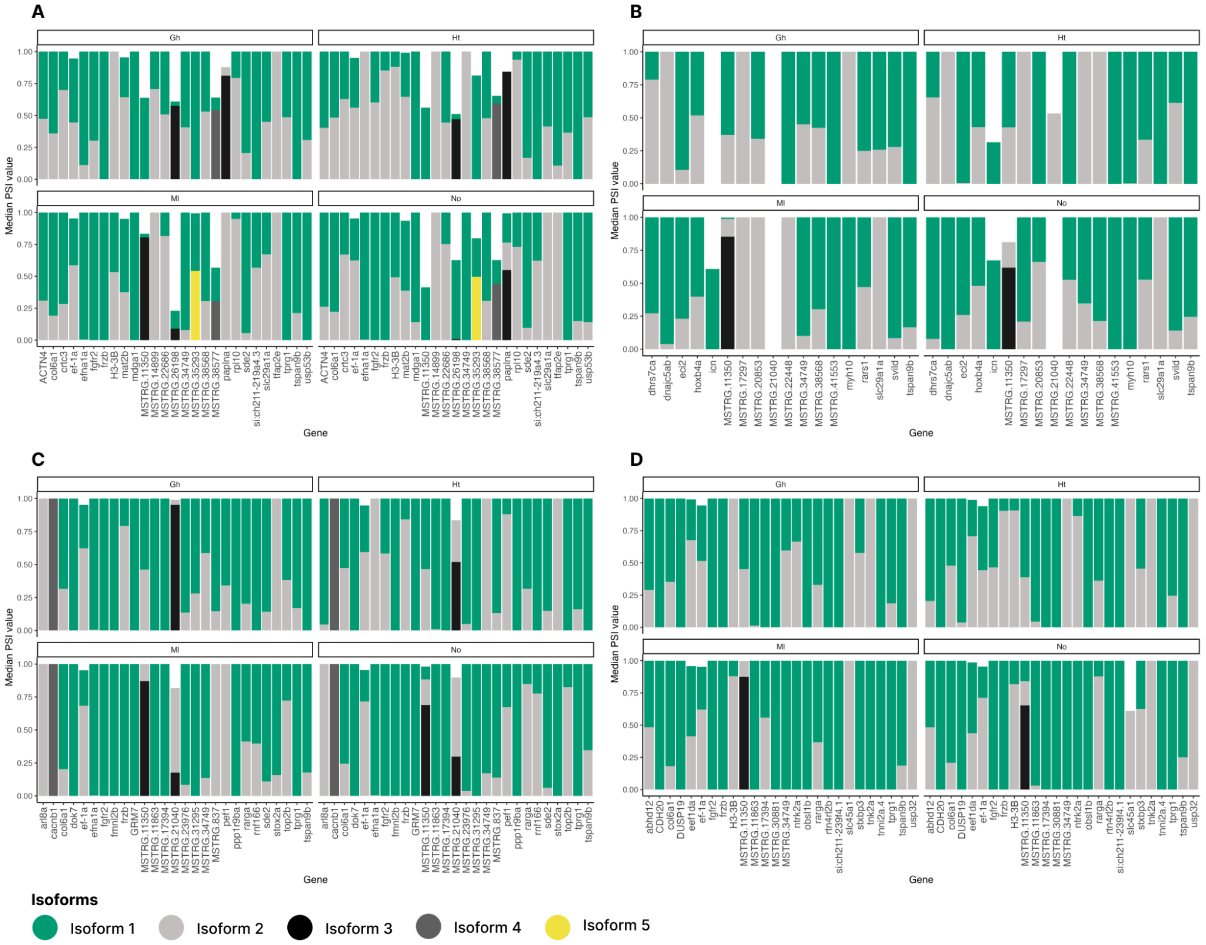

Fig. S7. *Stacked bar plots of isoforms with differential usage between at least one carnivore-herbivore comparison. Median PSI values (relative abundance) were calculated across 5 replicates each. Different shadings imply different isoforms: green (isoform 1), light grey (isoform 2), black (isoform 3), dark grey (isoform 4) and yellow (isoform 5). Barplots are shown for larval OJA (A), larval PJA (B), adult OJA (C) and adult PJA (D). Oral jaw apparatus (OJA), pharyngeal jaw apparatus (PJA), G. hiatus (Gh; carnivore), H. thereuterion (Ht; carnivore), M. lutea (Ml; herbivore) and N. omnicaeruleus (No; herbivore).*

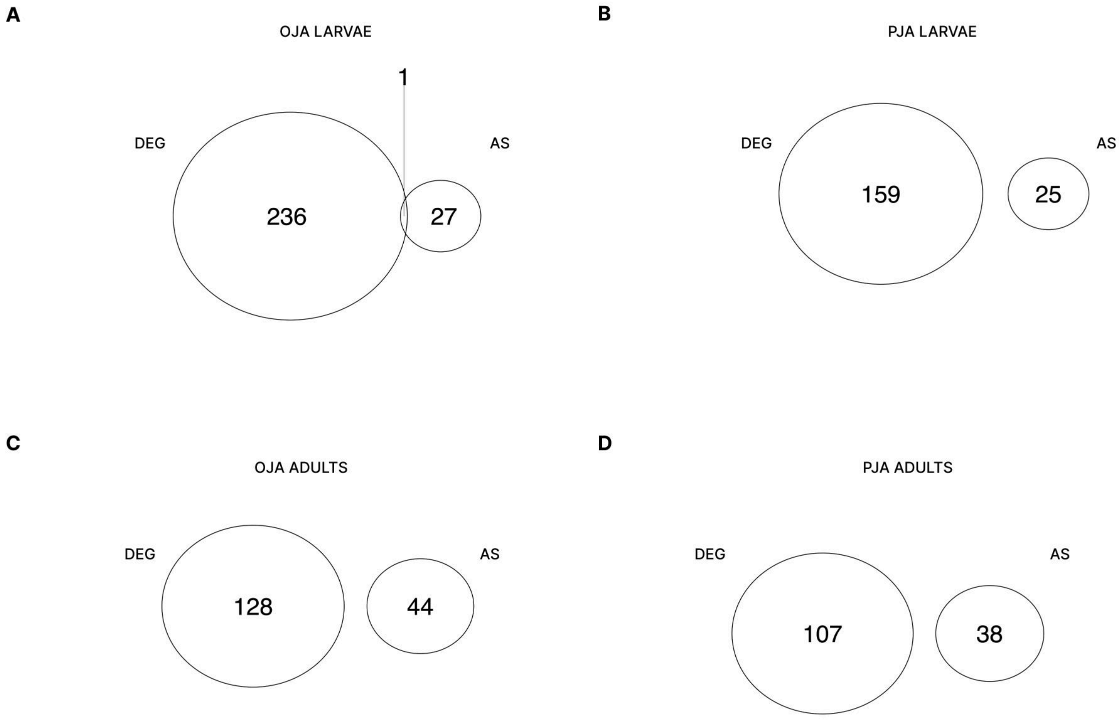

Fig. S8 *Overlap of differentially expressed genes (DEG) and alternatively spliced genes (AS) at the larval (L) or adult (A) stage in the oral jaw apparatus (OJA) and pharyngeal jaw apparatus (PJA) between herbivores and carnivores.*

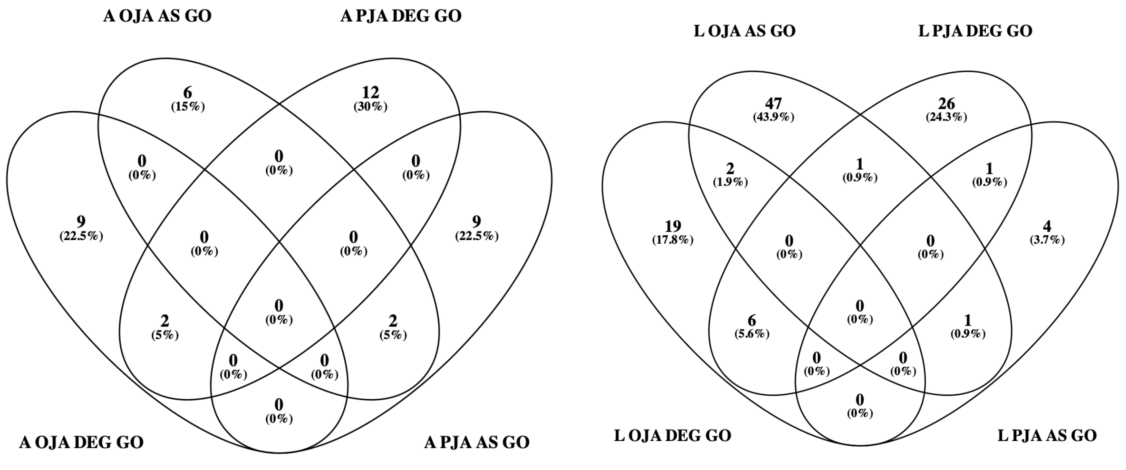

Fig. S9 *Overlap of Gene Ontology (GO) enrichment terms in the genes that were alternatively spliced (AS) or differentially expressed (DEG) at the larval (L) or adult (A) stage in the oral (OJA) and pharyngeal jaw apparatus (PJA).*

Supplementary analyses:

Gene expression modularity of the two jaws

Evolutionary modularity in gene regulation can contribute to morphological diversification and specialisation through the rewiring of gene regulatory networks (Wagner et al. 2007). We investigated gene expression differences between the oral and pharyngeal jaw apparatuses in herbivorous versus carnivorous species, to see whether the underlying gene regulatory architecture behind these functionally independent jaw modules differs between trophic niches. At stage 26 larvae, 1,889 (1768+121) genes were found to be differentially expressed between oral and pharyngeal jaw apparatus in all pairwise species comparisons, while at the adult stage only 209 (121+88) genes were consistently differentially expressed between the two jaws (Fig. S10 A and B; Fig. S11 A). One hundred and twenty-one of these genes were shared between both developmental stages, of which 68 were upregulated and 49 downregulated in the OJA versus PJA across all species and stages (Fig. S11 A). Four of these genes showed different directions of expression between the larval and the adult stage. Three of those four genes *ephb3*, *slc9a3* and *tbx3* were upregulated in larval PJA and downregulated in adult PJA. One gene, with the opposite expression pattern was unannotated. Several genes of the *Hox* family (*hoxa4a*, *hoxa4*, *hoxa5a*, *hoxb3a*, *hoxc5a*, *hoxc4a*) show a decreased expression in the OJA at both stages.

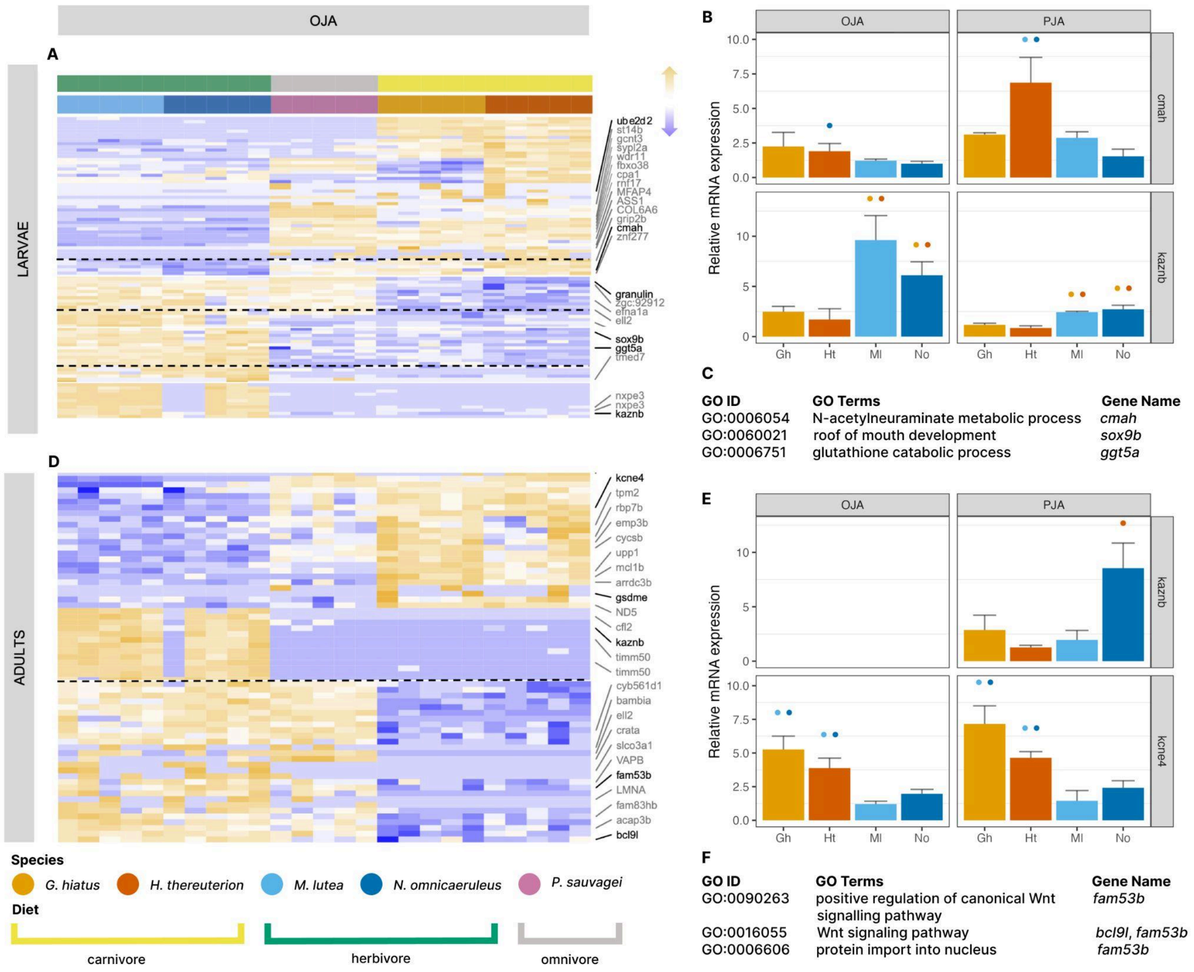

Fig. S10. *Bar plots show the number of differentially expressed genes between oral and pharyngeal jaw apparatus for each species in the larval (A) and adult (B) stage. The indicated line shows the number of genes that are shared across all species for each stage. Heatmaps show the gene expression pattern of 121 overlapping genes between the larval (C) and adult (D). Oral jaw apparatus (OJA), pharyngeal jaw apparatus (PJA),G. hiatus (Gh; bright orange; carnivore), H. thereuterion (Ht; dark orange; carnivore), M. lutea (Ml; bright blue; herbivore), N. omnicaeruleus (No; dark blue; herbivore) and P. sauvagei (Ps; pink; omnivore).*

GO enrichment of the 121 overlapping differentially expressed genes between the OJA and the PJA of all carnivores and herbivores for both life stages showed significant enrichment for genes involved in 'cranial skeletal system development', regulation of muscle contraction', 'multicellular organism development' and 'regulation of transcription, DNA-templated' (Fig. S11 B). Genes that were associated with the GO term 'cranial skeletal system development' include the genes *alx3, aristaless-like homeobox 3*, *prrx1a*, *paired related homeobox 1a*, *dlx4b*, *distal-less homeobox 4b* and *nkx3-2*, *NK3 homeobox 2* (Fig. 5 B).

A

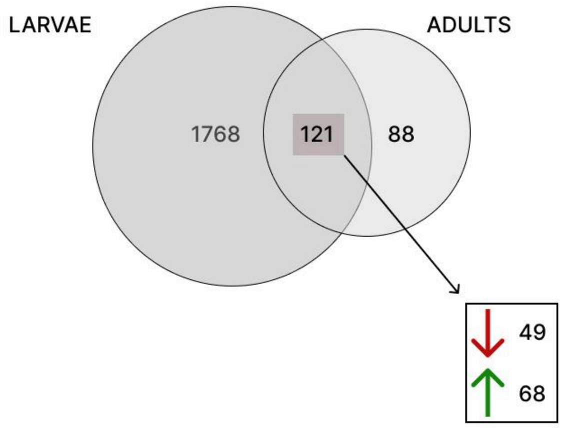

B

GO ID	GO Terms	Gene Name
GO:0001501	skeletal system development	*alx3, dlx4b, foxc1a, bglap, nkx3-2*
GO:1904888	cranial skeletal system development	*alx3, prrx1a, dlx4b, nkx3-2*
GO:0007275	multicellular organism development	*alx3*, hoxa4a, *hoxa4*, *hoxa5a, alx4a, prrx1a, hoxd4a, lama3, sfrp2, igfbp2a, angpt1, mb, dlx4b, foxc1a, desma, bglap, nkx3-2*

Figure S11: The Venn diagram shows the overlap of differentially expressed genes in the oral versus the pharyngeal jaw apparatus in larval and adults, and their direction of expression (upregulated genes are indicated by the upward facing arrow in green, downregulated genes by the downward facing arrow in red; direction indicated for OJA versus PJA) (A). Selected GO terms are listed in (B).

Developmental modularity is also reflected in the number of genes that are uniquely differentially expressed between oral and pharyngeal jaws at the larval vs adult stage (Figure S11). There are 1,768 genes that are only differentially expressed at the larval stage and 88 genes that are only expressed in adults, suggesting that these genes are performing unique functions during ontogeny - and that major differences in the two jaws are established early in development. Overall our results suggest that gene regulatory programs during jaw development are dynamic, controlling the basic development of jaw structures in larvae and later, in adults, shaping jaws to reflect species-specific trophic adaptations, presumably in response to environmental influences.

Candidate genes for cichlid jaw modularity

It has been proposed that evolutionary decoupling (i.e. modularity) of jaws played an important role in cichlid diversification (Liem 1973). However, this hypothesis has mostly been tested in form and function (Liem 1973; Hulsey et al. 2006; Conith and Albertson 2021), or in gene regulation at one developmental stage (Singh et al 2021). Conith and Albertson (2021) emphasised the importance of studying integration/modularity at different taxonomic and developmental levels to better understand its role in cichlid adaptive radiation. As mentioned above, many genes that differentiated OJA versus PJA were unique to each developmental stage. However, a subset of genes were differentially expressed between the two jaws at *both* stages across all species (a total of 121 genes, which represented only 6,8% of the shared genes across species for larvae but 63.7% for adults) and showed the same direction of expression (except four genes) (Figure 5). This points to a conserved function of these 121 genes in differentiating oral vs pharyngeal jaws of these 121 genes at both life stages. Several *Hox* genes were part of this conserved gene set as *Hox* genes play an important role in the anteroposterior patterning of organisms and are known to not be present in the first pharyngeal arch, the mandibular arch (MA), forming the jaws in gnathostomes (Kuratani 2004; Ahi 2016). Recently it has also been shown that genes of the *Hox* family are not only expressed during development but also in adult tissues, contributing to bone regeneration (Rux and Wellik 2017; Bradaschia-Correa et al. 2019).

Interestingly, we found that *alx3*, which is known to be involved in cranial skeletal system development (Khor and Ettensohn 2020) was also in this developmental conserved gene set, being upregulated in the oral jaws at both life stages. In zebrafish (*Danio rerio*), *alx* genes are enriched in frontonasal neural crest cells (NCC) at early stages of development, playing a role in shaping skeletal elements of the anterior neurocranium (Mitchell et al. 2021) . While *Alx3* has been repeatedly lost in chicken (*Gallus gallus*), a frog (*Xenopus tropicalis*) and lizard (*Anolis carolinensis*) species, its role might be compensated for by *Alx1* and/or *Alx4* (McGonnell et al. 2011). An alx1 haplotype has been associated with beak shape diversification across the adaptive radiation of Darwin's finches (Lamichhaney et al. 2015). Similar to cichlid jaws, beak shape in finches has evolved in response to selection pressures to adapt to different food sources. Our findings suggest the ALX transcription factors

may play a conserved function in craniofacial and trophic diversification during adaptive radiation across vertebrates.

References

Ahi EP. 2016 Signalling pathways in trophic skeletal development and morphogenesis: Insights from studies on teleost fish. *Dev. Biol. J*. **420**(1), 11–31. (doi: 10.1016/j.ydbio.2016.10.003)

Bradaschia-Correa V, Leclerc K, Josephson AM, Lee S, Palma L, Litwa HP, Neibart SS, Huo JC, Leucht P. 2019 Hox gene expression determines cell fate of adult periosteal stem/progenitor cells. *Sci. Rep*. **9**(1), 1–13. (doi: 10.1038/s41598-019-41639-7)

Conith AJ, Albertson RC. 2021 The cichlid oral and pharyngeal jaws are evolutionarily and genetically coupled. *Nat. Commun*. **12**(1), 1–11. (doi: 10.1038/s41467-021-25755-5)

Hulsey CD, García de León FJ, Rodiles-Hernández R. 2006 Micro- and macroevolutionary decoupling of cichlid jaws: a test of Liem's key innovation hypothesis. *Evolution*. **60**(10), 2096–2109. (doi: 10.1111/j.0014-3820.2006.tb01847.x)

Khor JM, Ettensohn CA. 2020 Transcription Factors of the Alx Family: Evolutionarily Conserved Regulators of Deuterostome Skeletogenesis. *Front. Genet*. **11**, 569314. (doi: 10.3389/fgene.2020.569314)

Kuratani S. 2004 Evolution of the vertebrate jaw: Comparative embryology and molecular developmental biology reveal the factors behind evolutionary novelty. *J. Anat*. **205**(5), 335–347. (doi: 10.1111/j.0021-8782.2004.00345.x)

Lamichhaney S, Berglund J, Almén MS, Maqbool K, Grabherr M, Martinez-Barrio A, Promerová M, Rubin CJ, Wang C, Zamani N, Grant BR, Grant PR, Webster MT, Andersson L. 2015 Evolution of Darwin's finches and their beaks revealed by genome sequencing. *Nature*. **518**(7539), 371–375. (doi: 10.1038/nature14181)

Liem KF. 1973 Evolutionary Strategies and Morphological Innovations: Cichlid Pharyngeal Jaws. *Syst. Zool.* **22**(4), 425. (doi: 10.2307/2412950)

McGonnell IM, Graham A, Richardson J, Fish JL, Depew MJ, Dee CT, Holland PWH, Takahashi T. 2011 Evolution of the Alx homeobox gene family: Parallel retention and independent loss of the vertebrate Alx3 gene. *Evol. Dev*. **13**(4), 343–351. (doi: 10.1111/j.1525-142X.2011.00489.x)

Mitchell JM, Sucharov J, Pulvino AT, Brooks EP, Gillen AE, Nichols JT. 2021 The alx3 gene shapes the zebrafish neurocranium by regulating frontonasal neural crest cell differentiation timing. *Development*. **148**(7), dev197483. (doi: 10.1242/dev.197483)

Rux DR, Wellik DM. 2017 Hox genes in the adult skeleton: Novel functions beyond embryonic development. *Dev. Dynam*. **246**(4), 310–317. (doi: 10.1002/dvdy.24482)

Singh P, Ahi EP, Sturmbauer C. 2021 Gene coexpression networks reveal molecular interactions underlying cichlid jaw modularity. *BMC Ecol. Evol*. **21**(1), 62. (doi: 10.1186/s12862-021-01787-9)

Wagner GP, Pavlicev M, Cheverud JM. 2007 The road to modularity. *Nat. Rev. Genet*. 8(12), 921–931. (doi: 10.1038/nrg2267)

Visual check for batch effect:

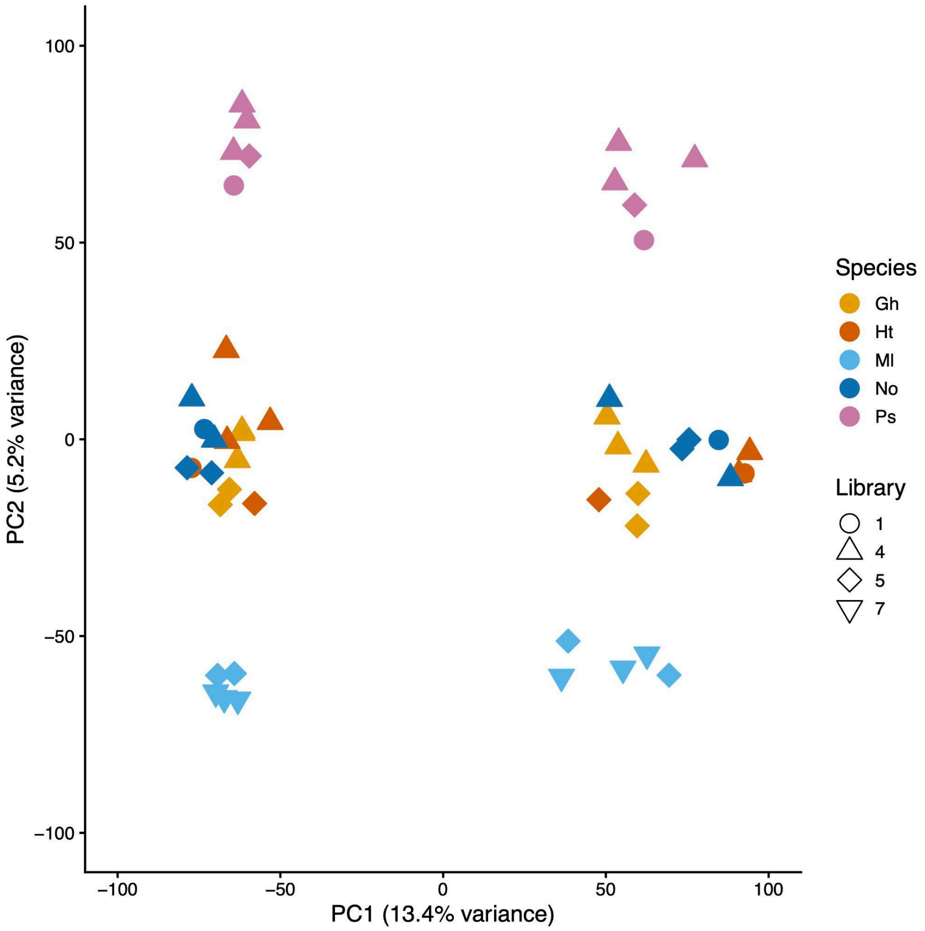

Figure S12: Normalised gene expression PCA plot for larvae samples based on 37,334 genes to confirm that no batch effect was introduced via library prep/ sequencing. PCA annotations: Library: Different libraries for sequencing that the samples were put on; Species: G. hiatus (Gh; bright orange; carnivore), H. thereuterion (Ht; dark orange; carnivore), M. lutea (Ml; bright blue; herbivore), N. omnicaeruleus (No; dark blue; herbivore) and P. sauvagei (Ps; pink; omnivore).

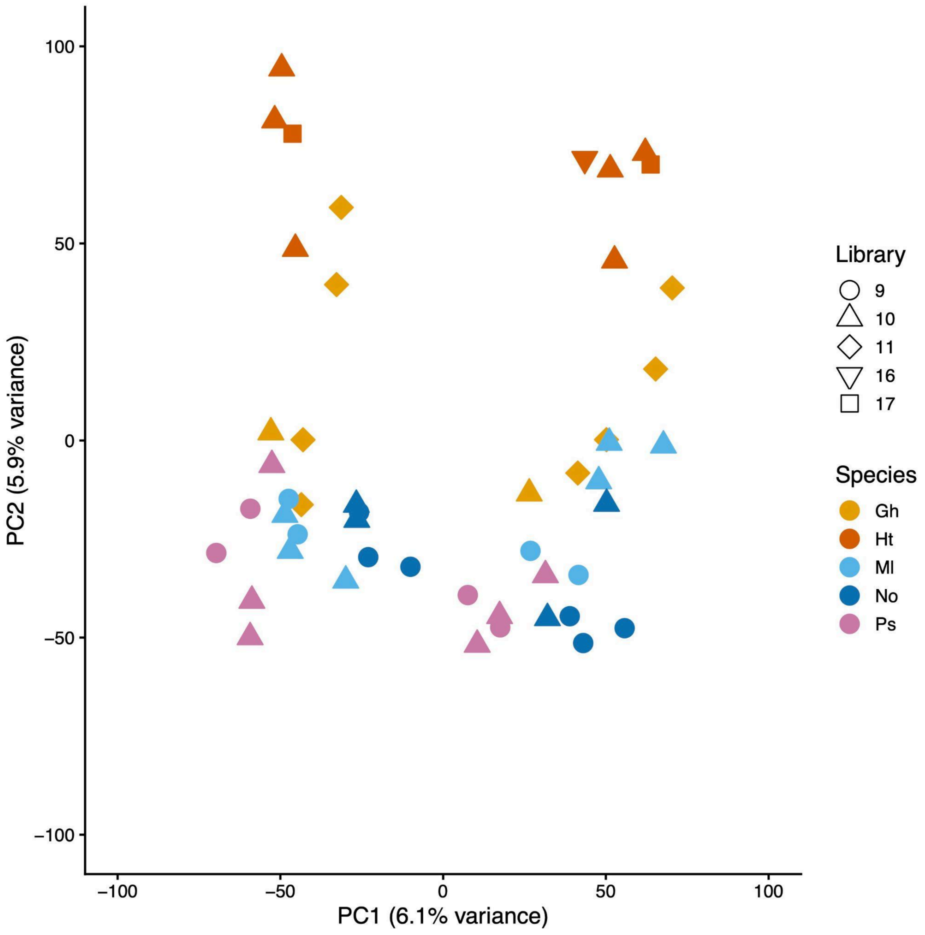

Figure 13: Normalised gene expression PCA plot for adult samplesvbased on 37,334 genes to confirm that no batch effect was introduced via library prep/ sequencing. PCA annotations: Library: Different libraries for sequencing that the samples were put on; Species: G. hiatus (Gh; bright orange; carnivore), H. thereuterion (Ht; dark orange; carnivore), M. lutea (Ml; bright blue; herbivore), N. omnicaeruleus (No; dark blue; herbivore) and P. sauvagei (Ps; pink; omnivore).

Volcano plots: Adults

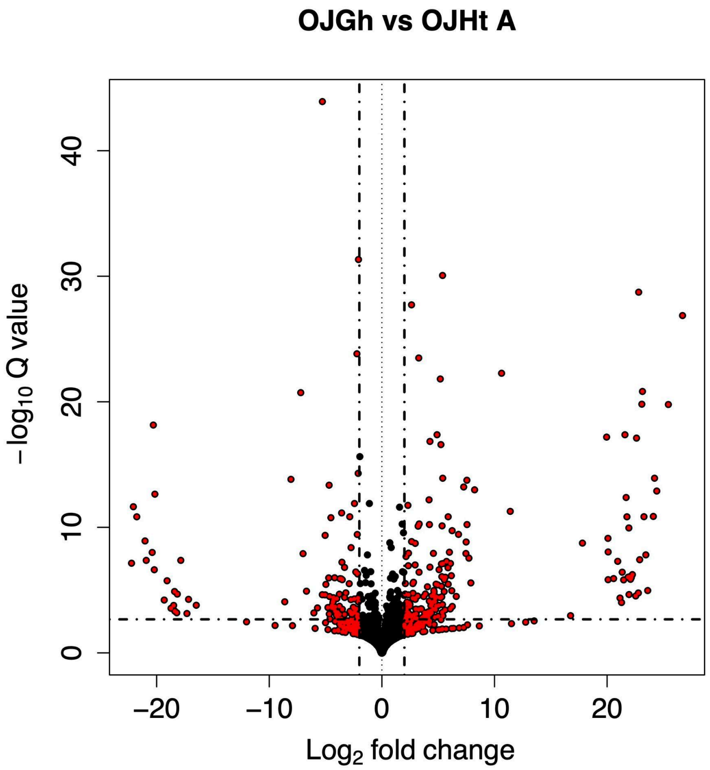

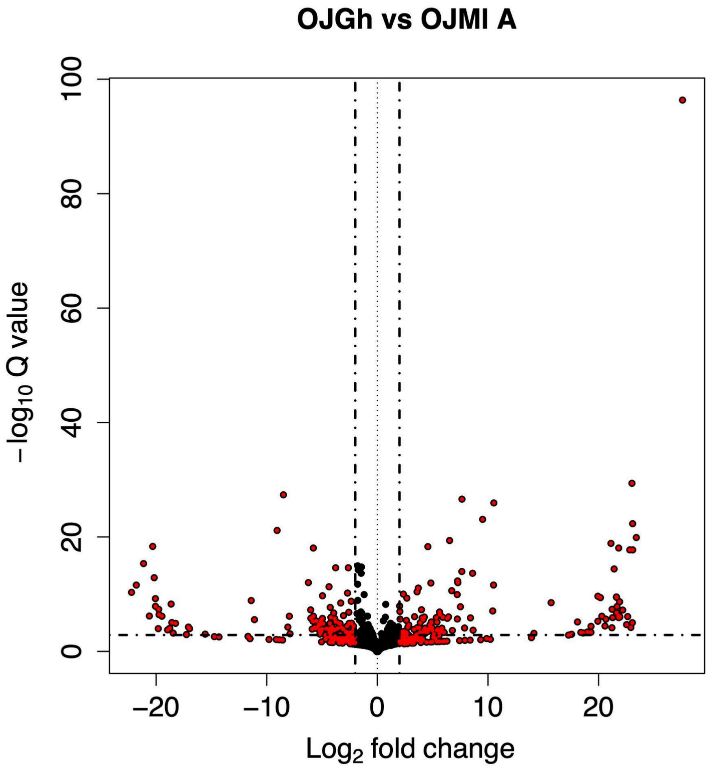

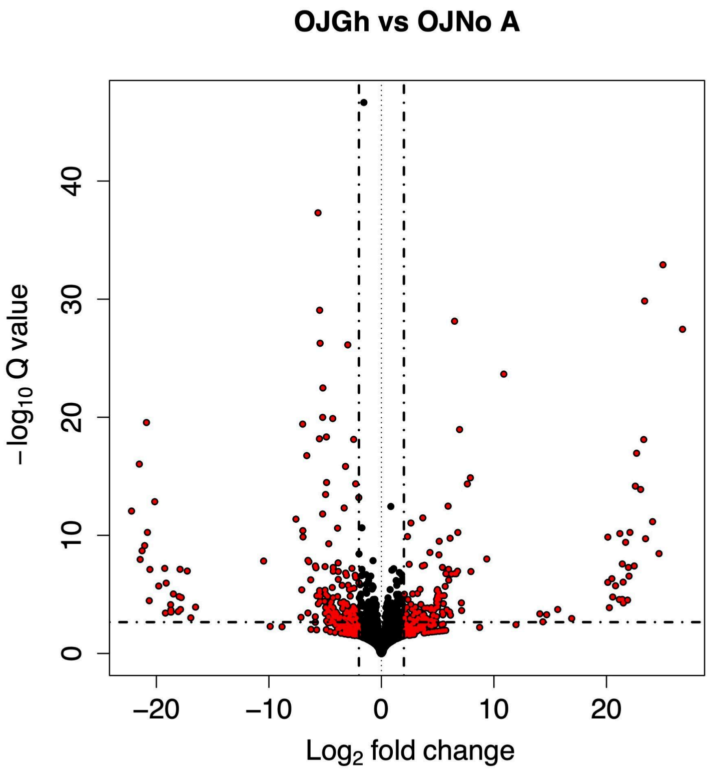
OJGh vs OJNo A
−log10 Q value
Log2 fold change
0
10
20
30
40
−20
−10
0
10
20

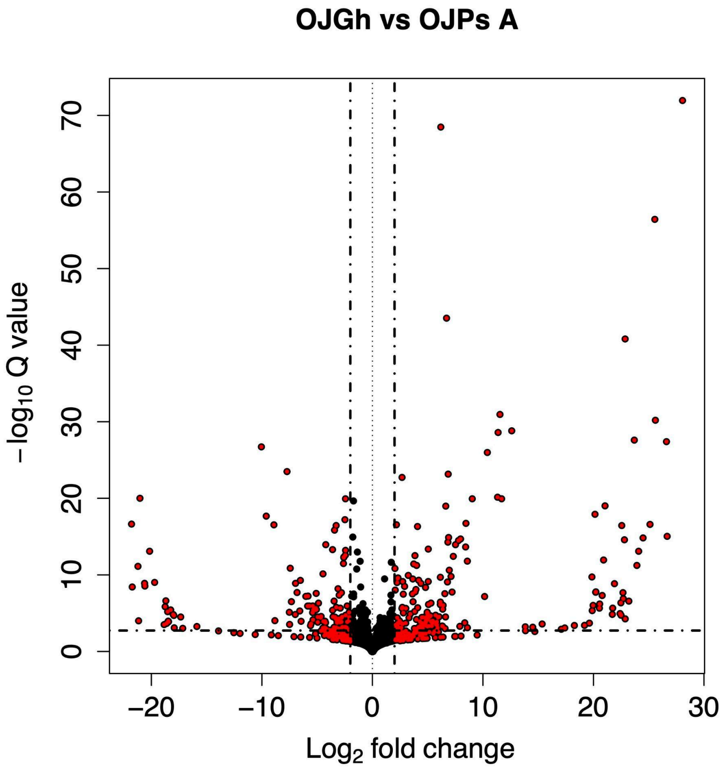
OJGh vs OJPs A
−log10 Q value
Log2 fold change
0
10
20
30
40
50
60
70
−20
−10
0
10
20
30

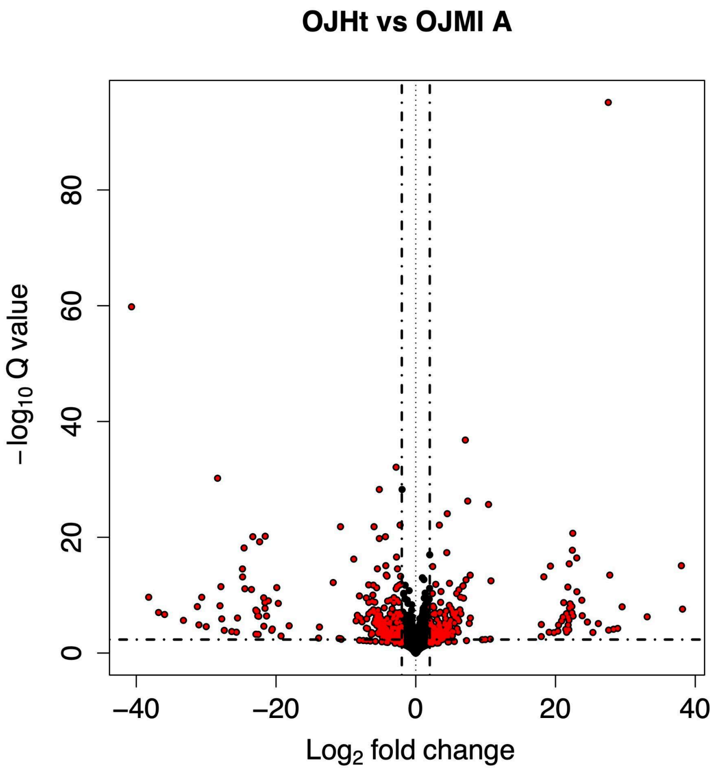
OJHt vs OJMI A
−log10 Q value
Log2 fold change
0
20
40
60
80
−40
−20
0
20
40

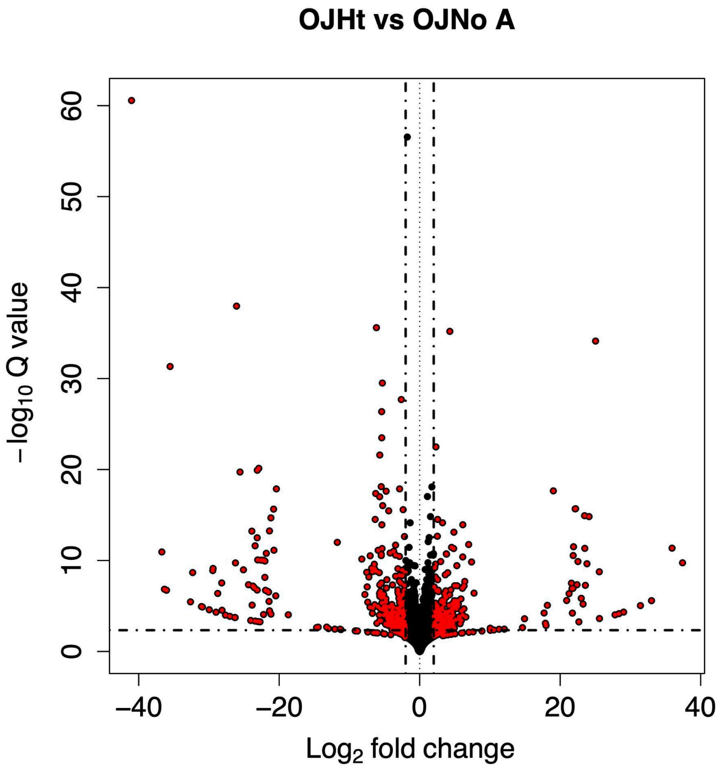
OJHt vs OJNo A
−log10 Q value
Log2 fold change
0
10
20
30
40
50
60
−40
−20
0
20
40

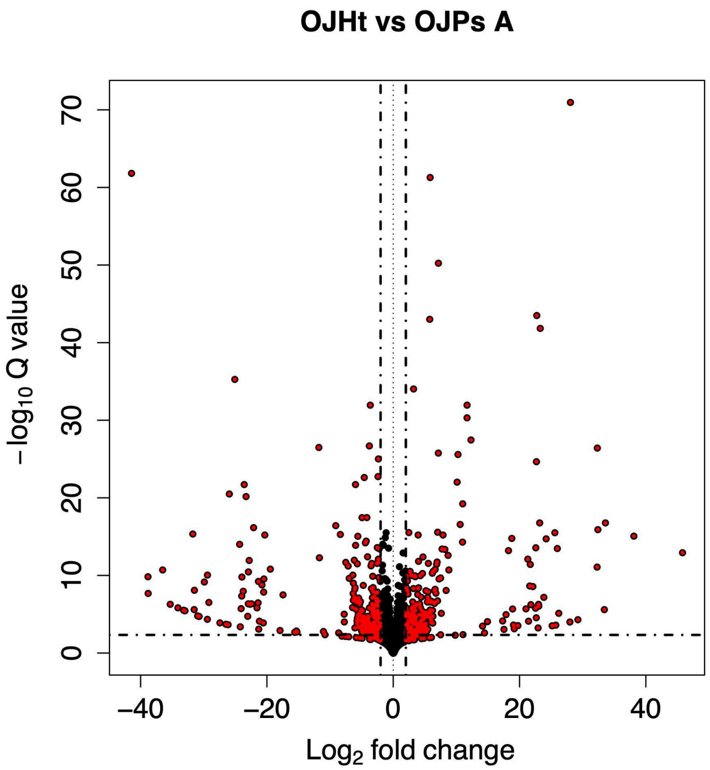
OJHt vs OJPs A
−log10 Q value
0
10
20
30
40
50
60
70
−40
−20
0
20
40
Log2 fold change

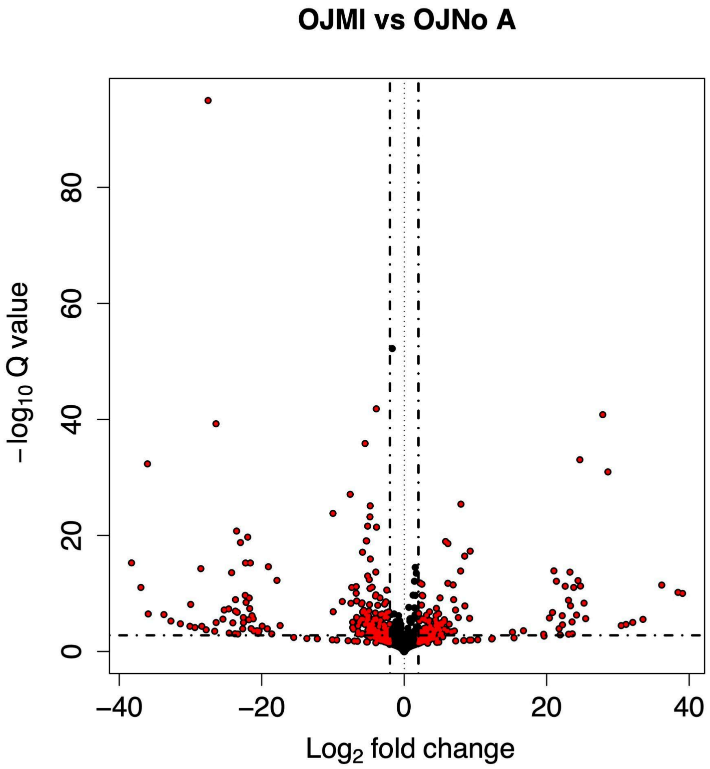
OJMl vs OJNo A
−log10 Q value
0
20
40
60
80
−40
−20
0
20
40
Log2 fold change

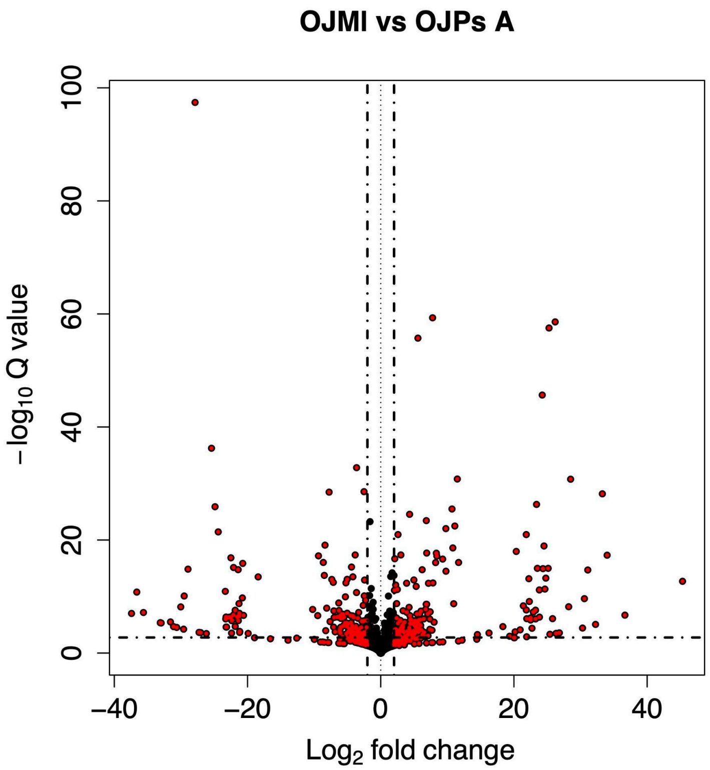
OJMl vs OJPs A
−log10 Q value
Log2 fold change
100
80
60
40
20
0
−40
−20
0
20
40

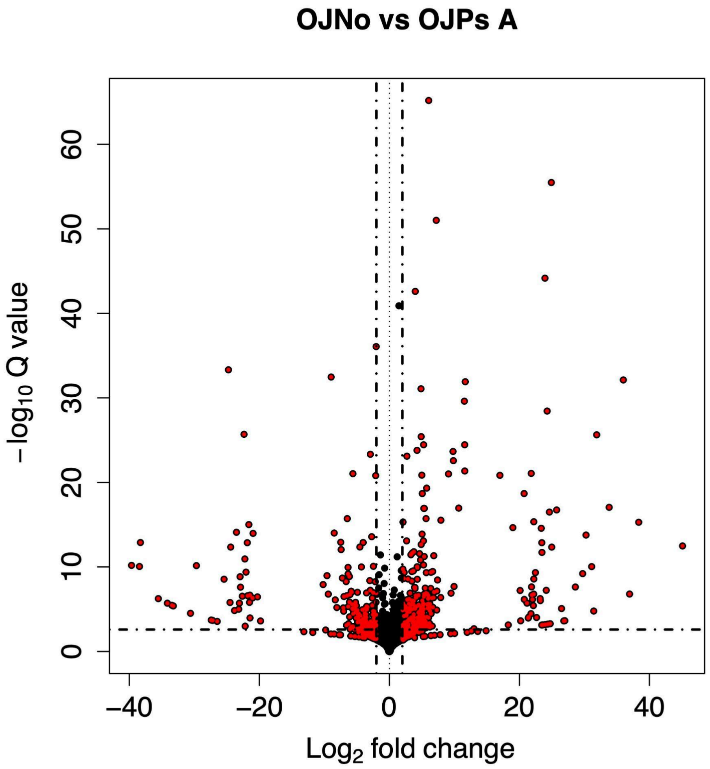
OJNo vs OJPs A
−log10 Q value
Log2 fold change
60
50
40
30
20
10
0
−40
−20
0
20
40

PJGh vs PJHt A

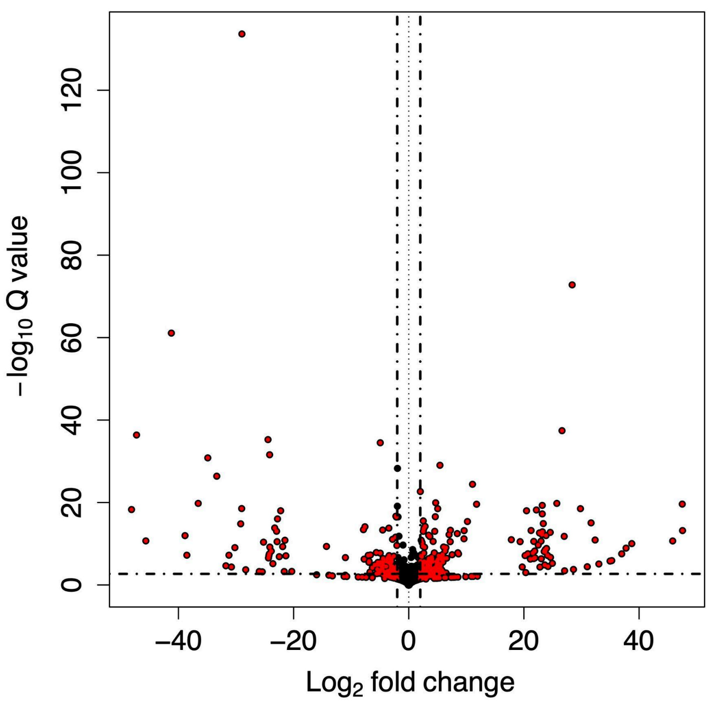

PJGh vs PJMl A

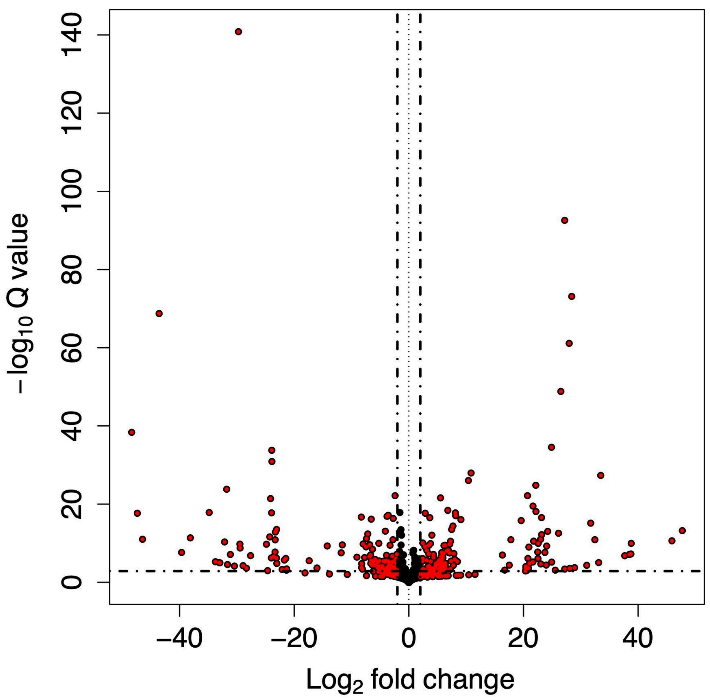

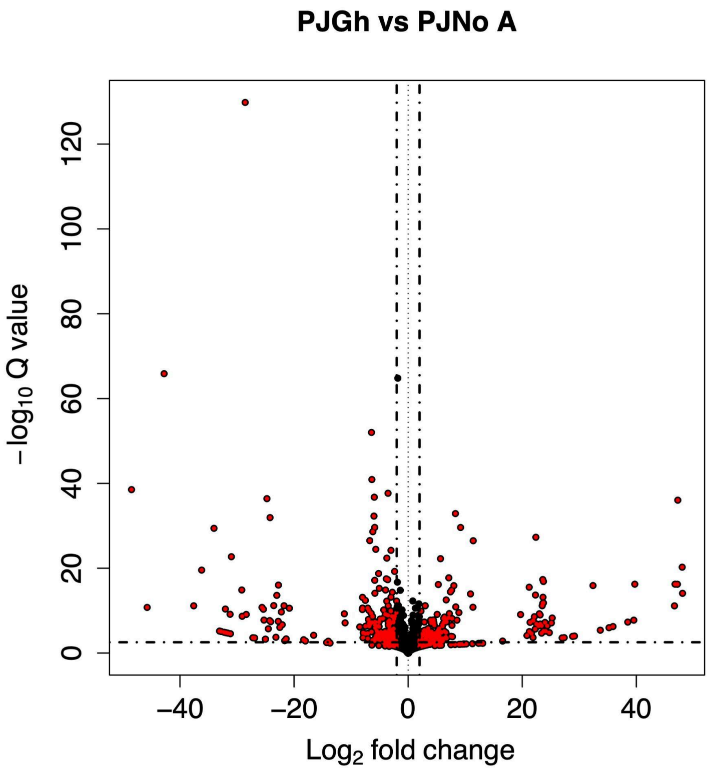
PJGh vs PJNo A
−log10 Q value
0
20
40
60
80
100
120
−40
−20
0
20
40
Log2 fold change

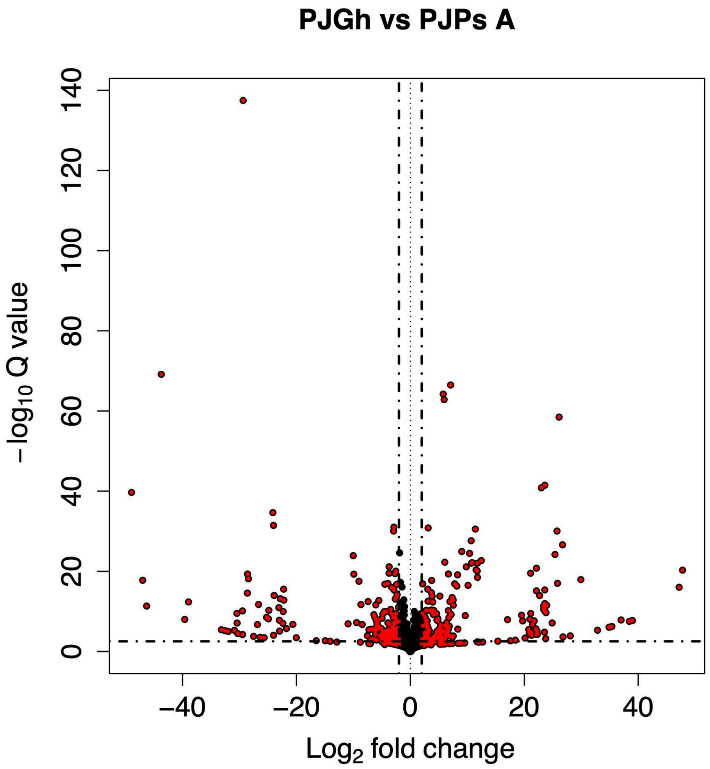
PJGh vs PJPs A
−log10 Q value
0
20
40
60
80
100
120
140
−40
−20
0
20
40
Log2 fold change

PJHt vs PJMI A

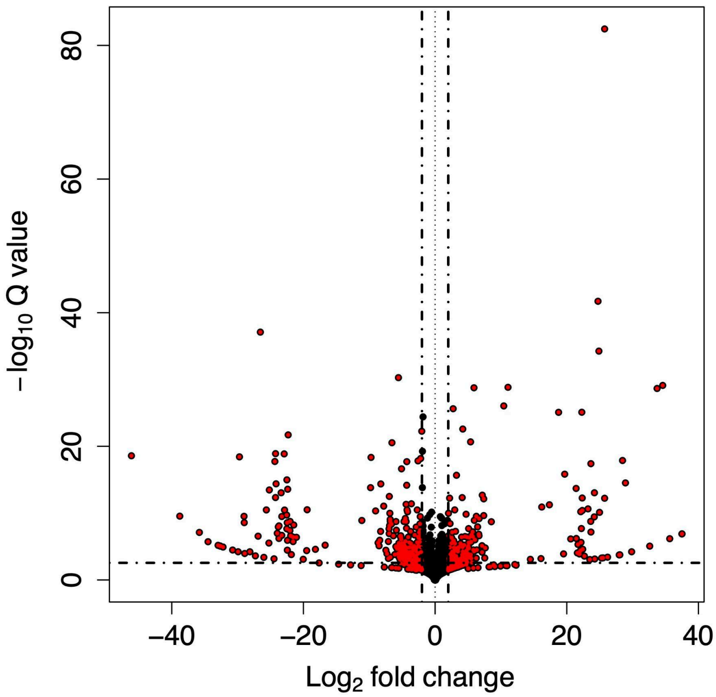

PJHt vs PJNo A

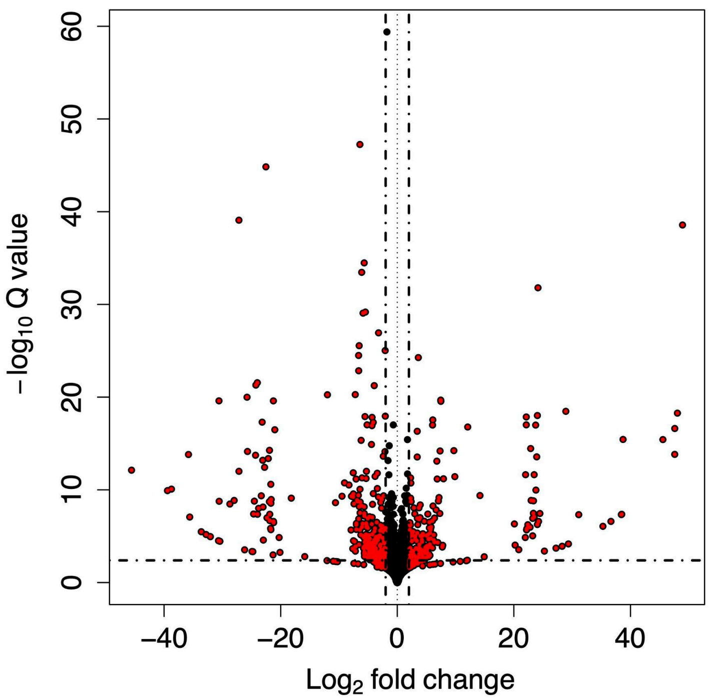

PJHt vs PJPs A

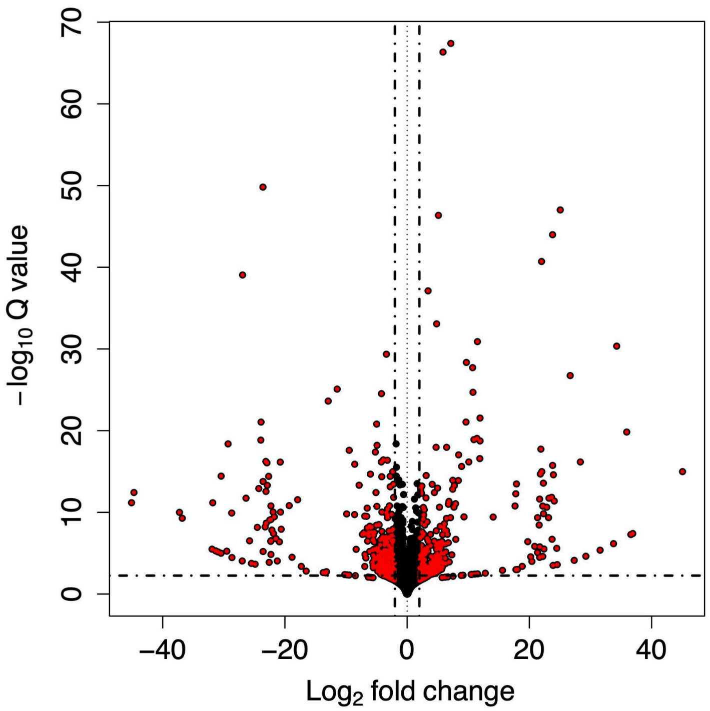

PJMl vs PJNo A

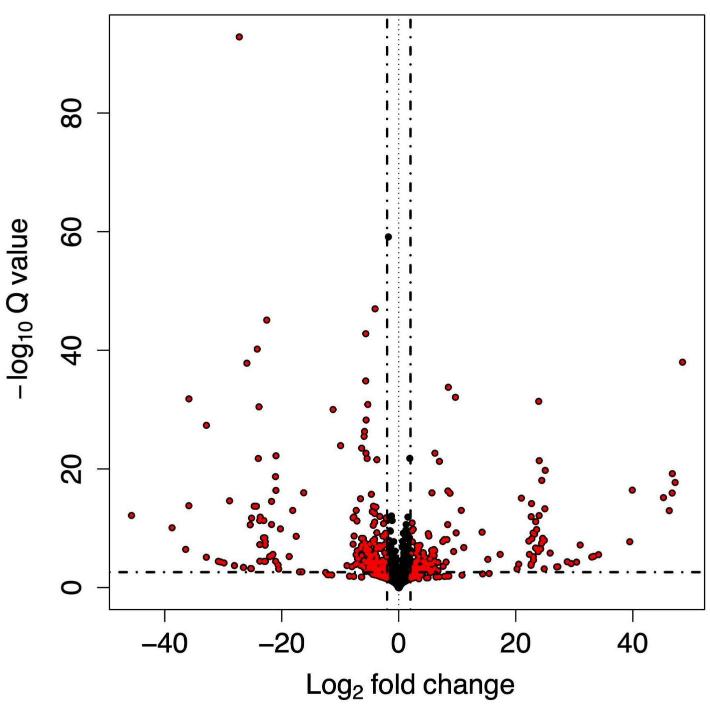

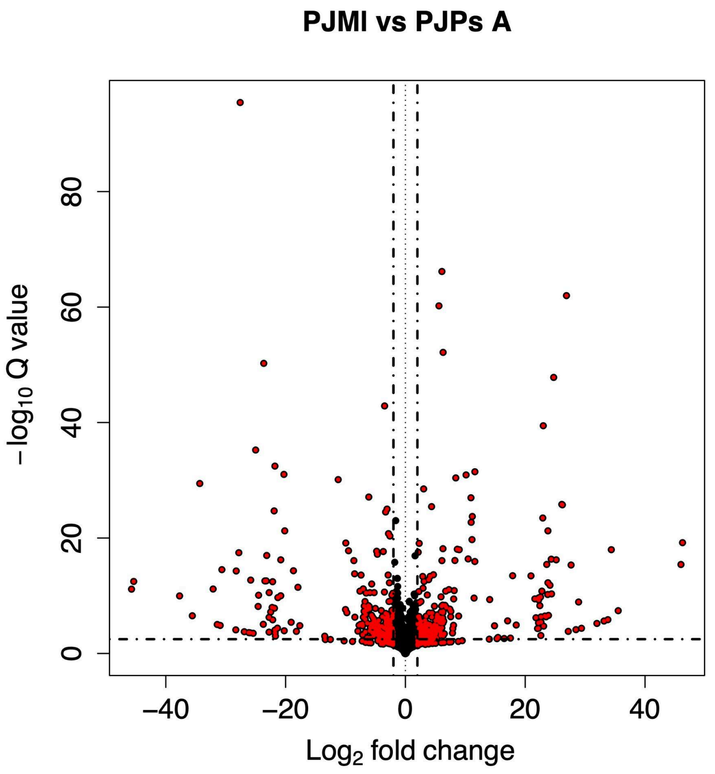
PJMl vs PJPs A
80
60
40
20
0
−log10 Q value
−40
−20
0
20
40
Log2 fold change

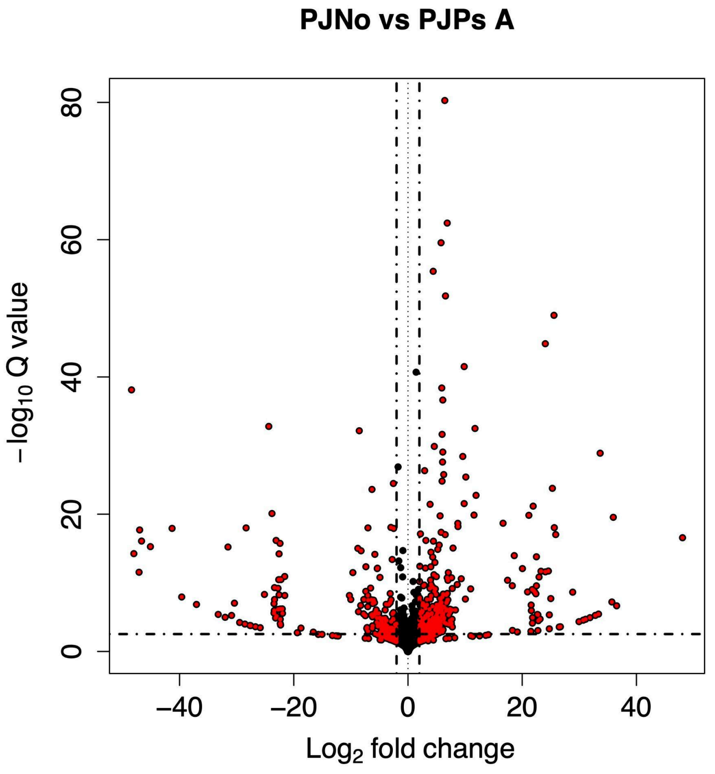
PJNo vs PJPs A
80
60
40
20
0
−log10 Q value
−40
−20
0
20
40
Log2 fold change

Larvae:

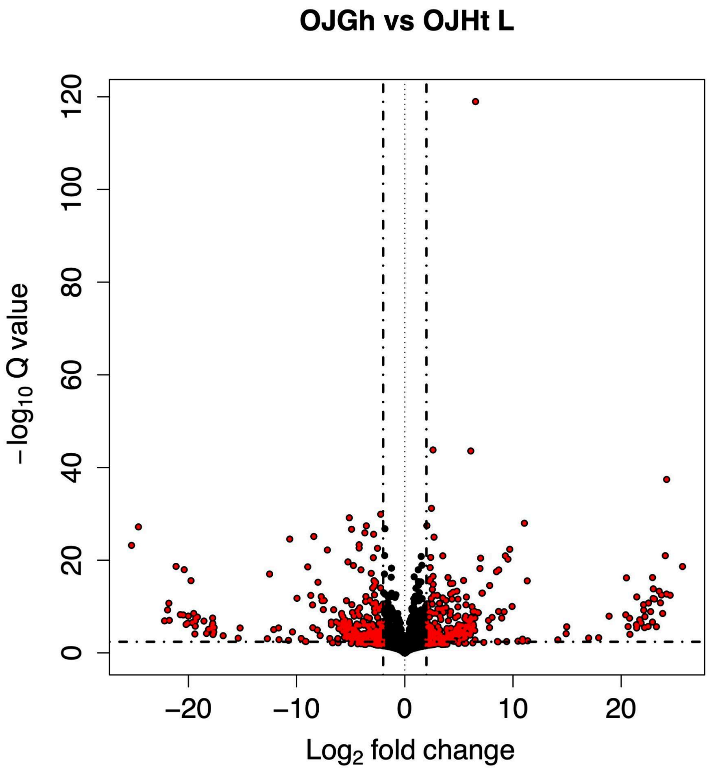
OJGh vs OJHt L
0
20
40
60
80
100
120
−log10 Q value
−20
−10
0
10
20
Log2 fold change

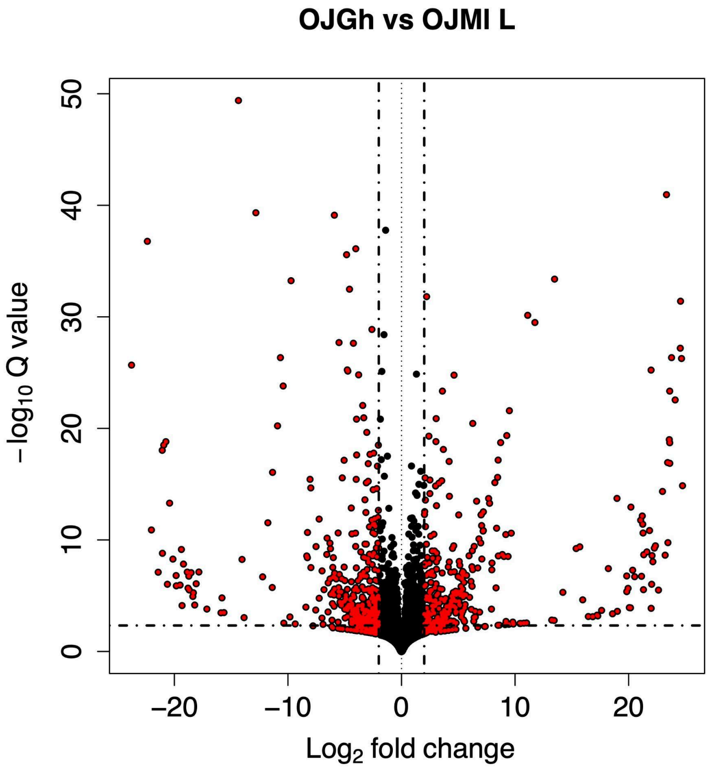
OJGh vs OJMl L
0
10
20
30
40
50
−log10 Q value
−20
−10
0
10
20
Log2 fold change

OJGh vs OJNo L

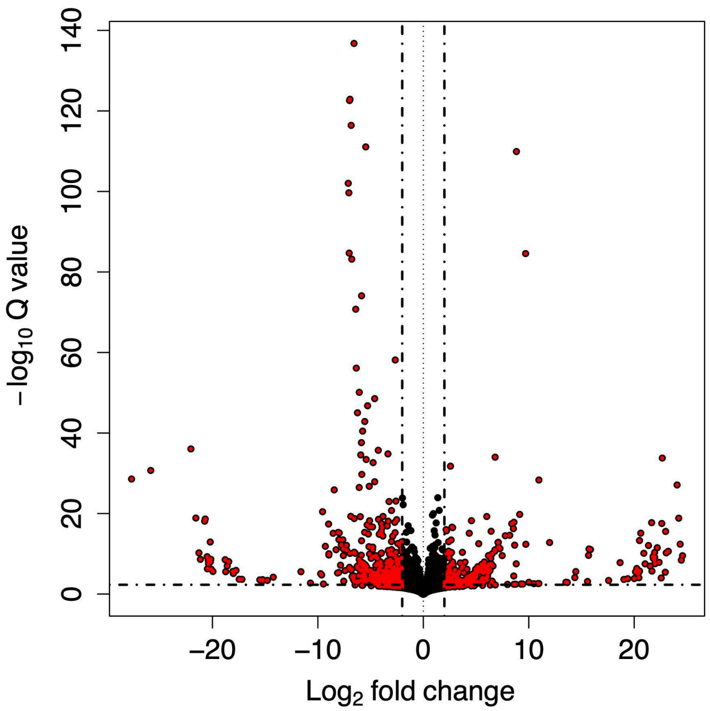

OJGh vs OJPs L

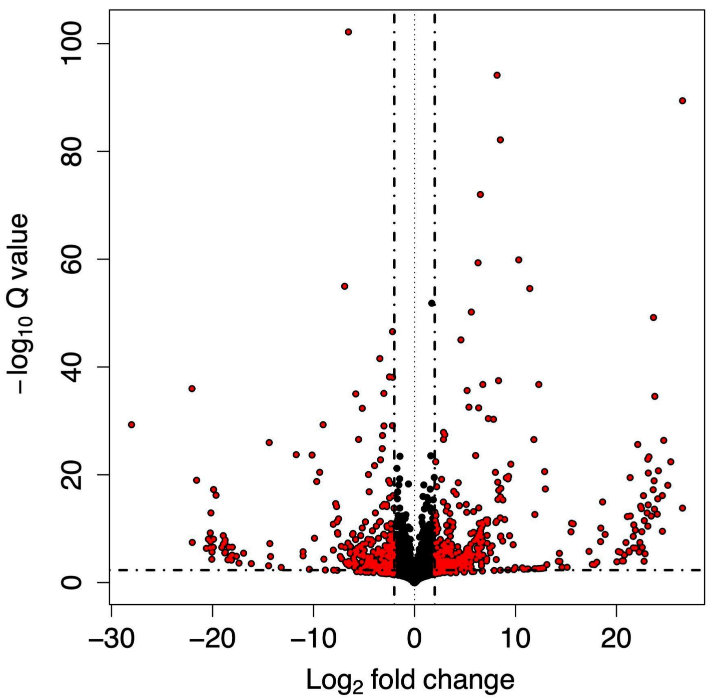

OJHt vs OJMI L

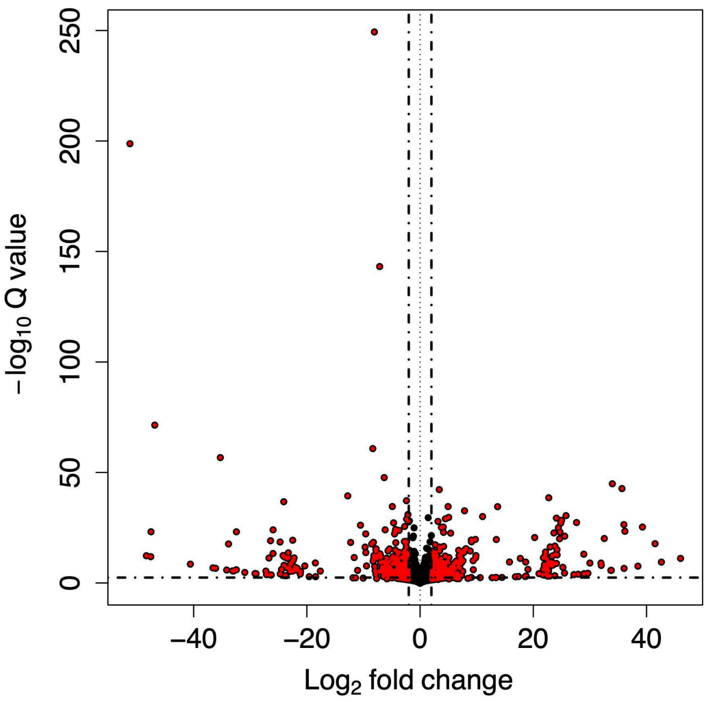

OJHt vs OJNo L

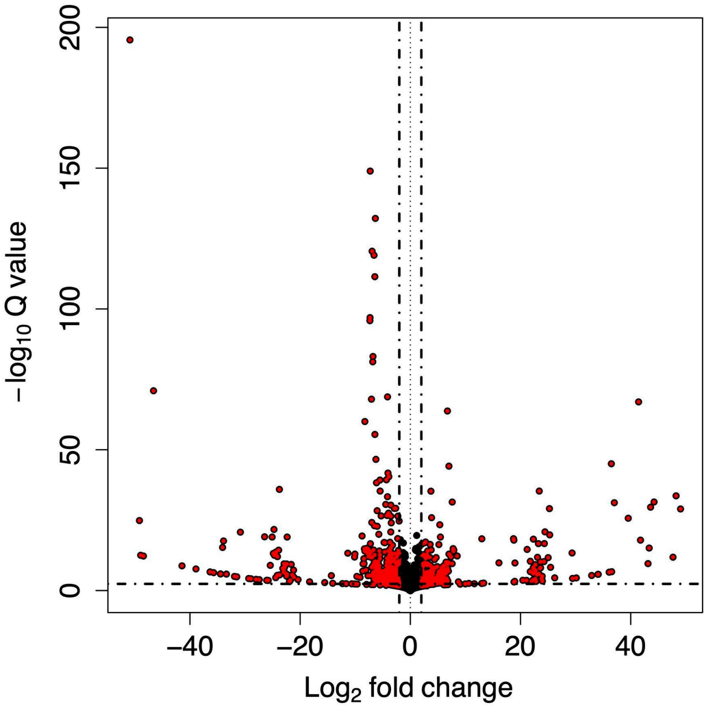

OJHt vs OJPs L

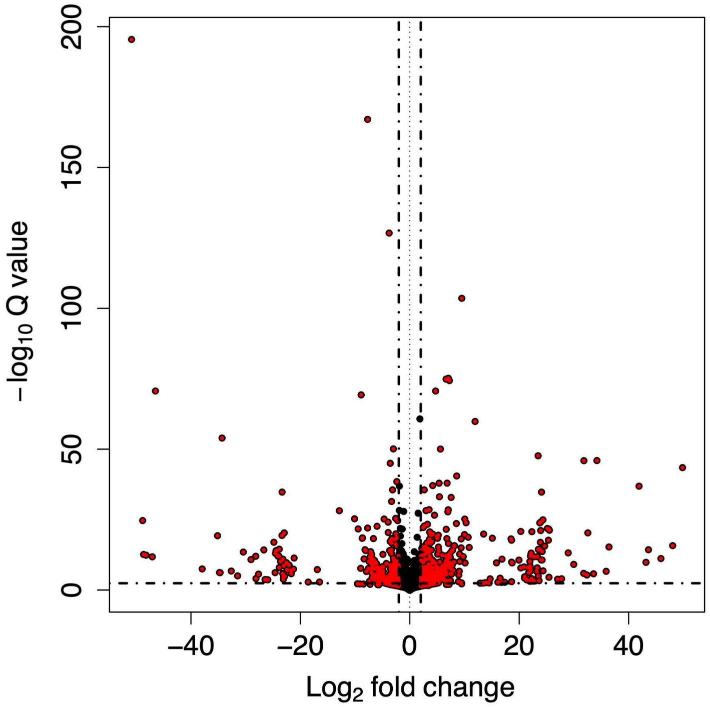

OJMl vs OJNo L

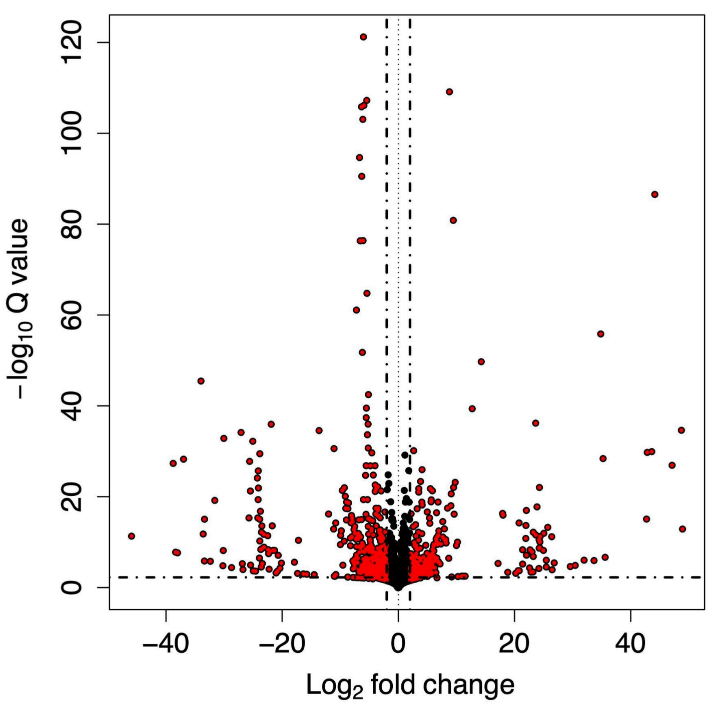

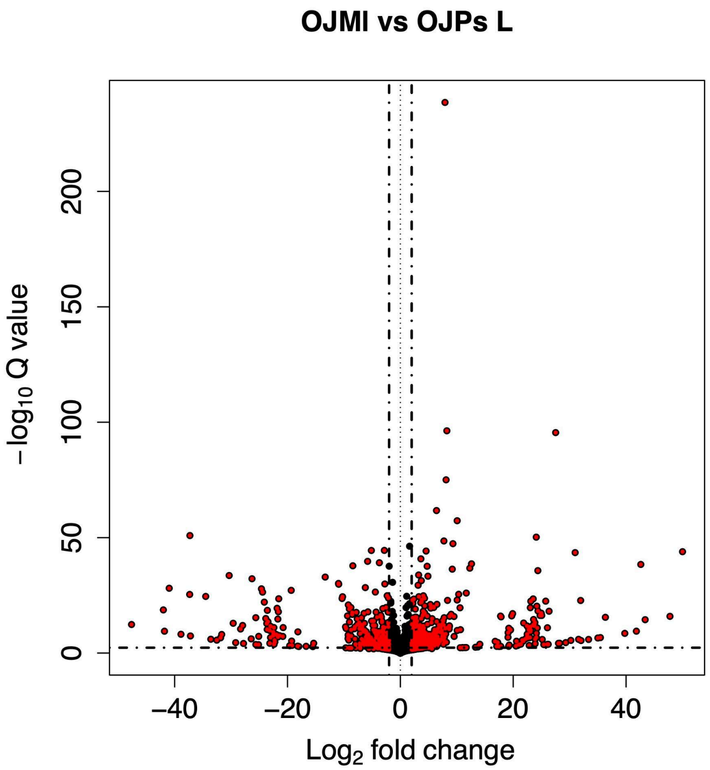
OJMl vs OJPs L
−log10 Q value
0
50
100
150
200
−40
−20
0
20
40
Log2 fold change

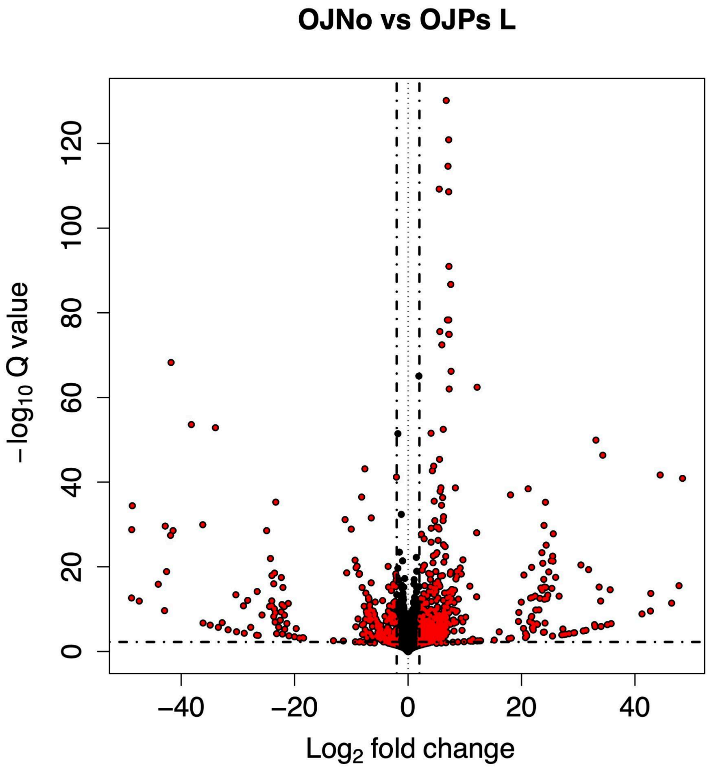
OJNo vs OJPs L
−log10 Q value
0
20
40
60
80
100
120
−40
−20
0
20
40
Log2 fold change

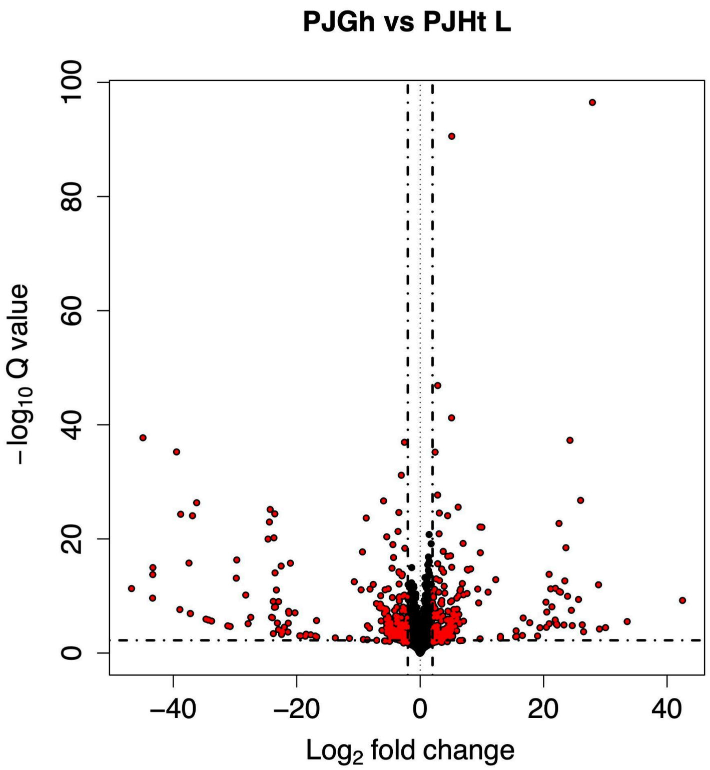
PJGh vs PJHt L
−log10 Q value
0
20
40
60
80
100
−40
−20
0
20
40
Log2 fold change

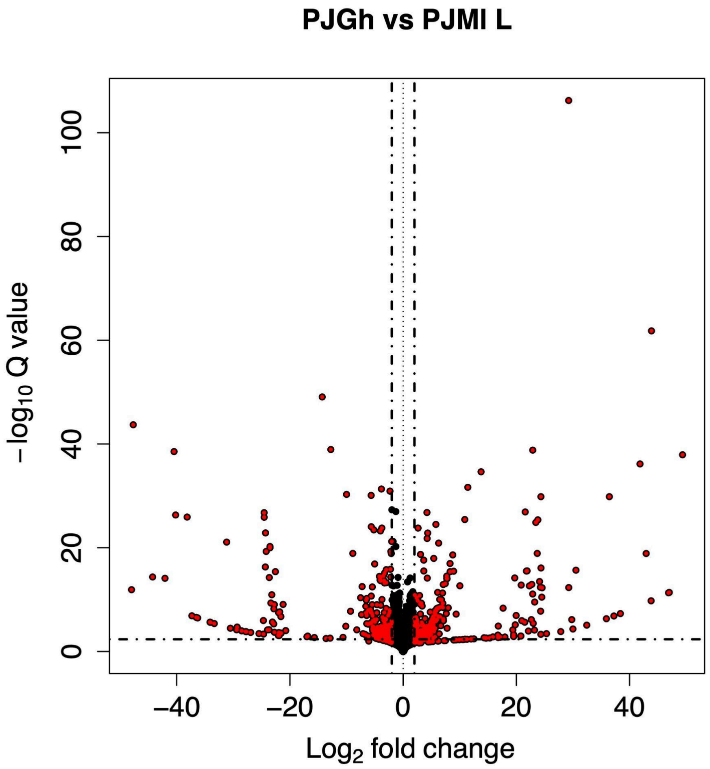
PJGh vs PJMl L
−log10 Q value
0
20
40
60
80
100
−40
−20
0
20
40
Log2 fold change

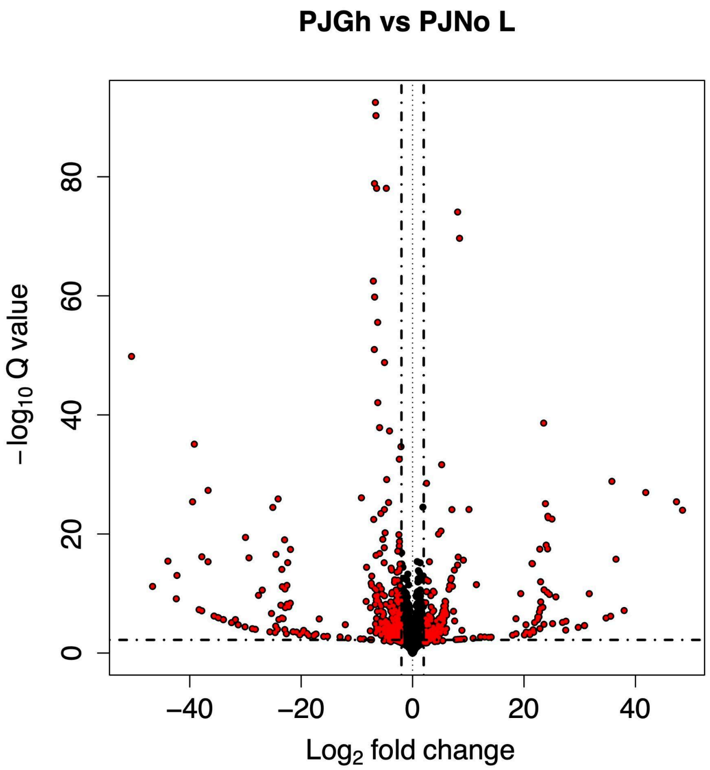

PJGh vs PJNo L
−log10 Q value
0
20
40
60
80
−40
−20
0
20
40
Log2 fold change

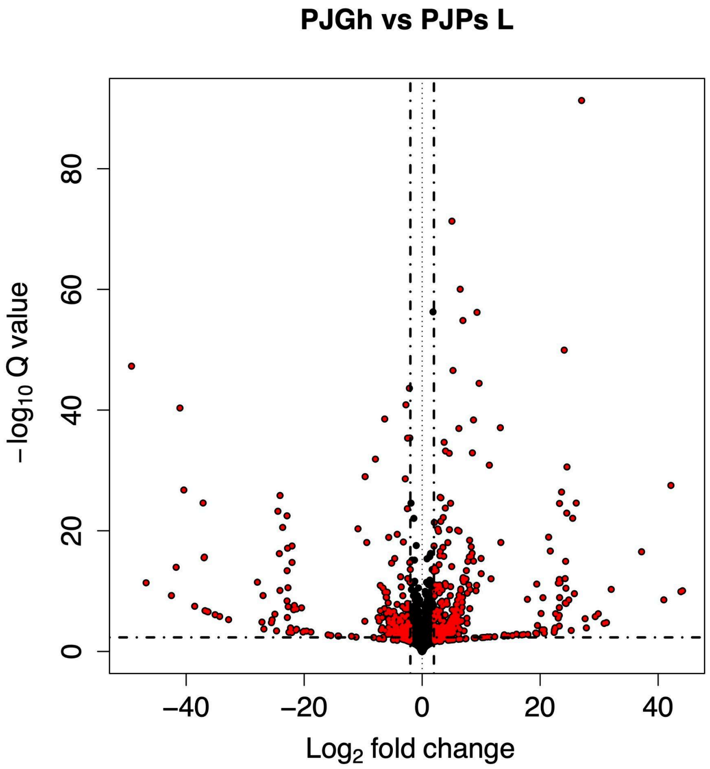

PJGh vs PJPs L
−log10 Q value
0
20
40
60
80
−40
−20
0
20
40
Log2 fold change

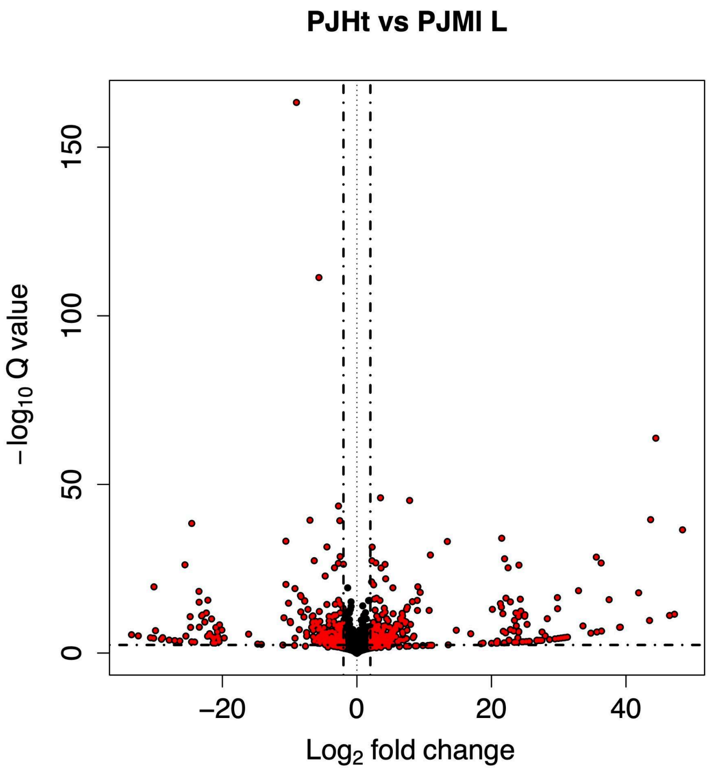
PJHt vs PJMI L
−log10 Q value
0
50
100
150
−20
0
20
40
Log2 fold change

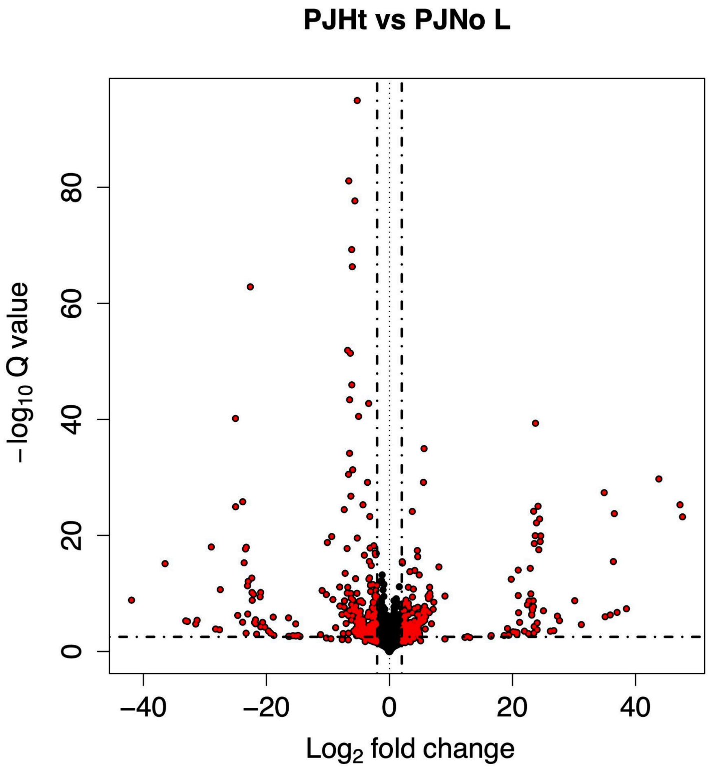
PJHt vs PJNo L
−log10 Q value
0
20
40
60
80
−40
−20
0
20
40
Log2 fold change

PJHt vs PJPs L

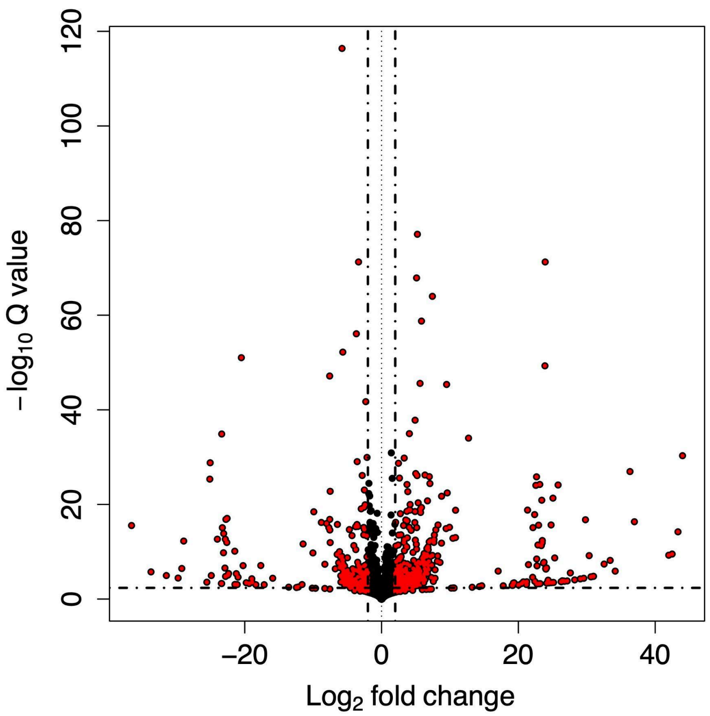

PJMl vs PJNo L

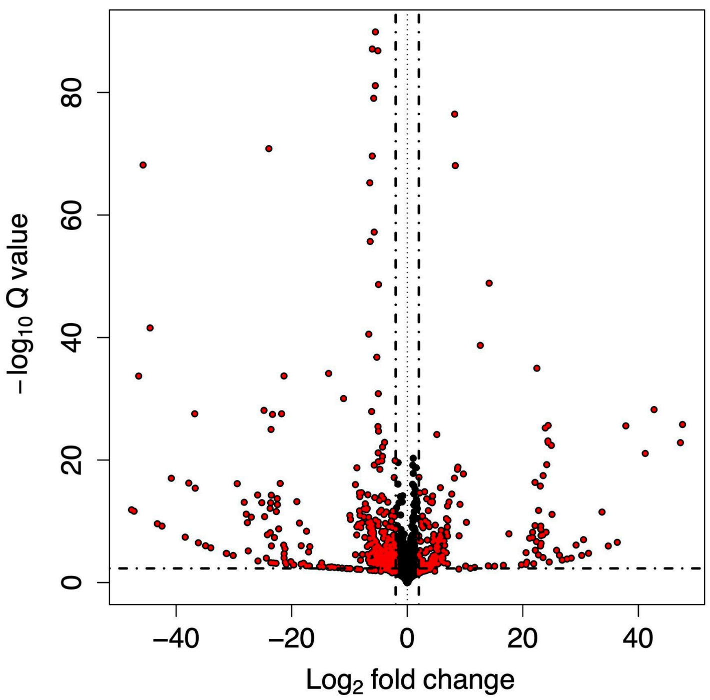

PJMl vs PJPs L

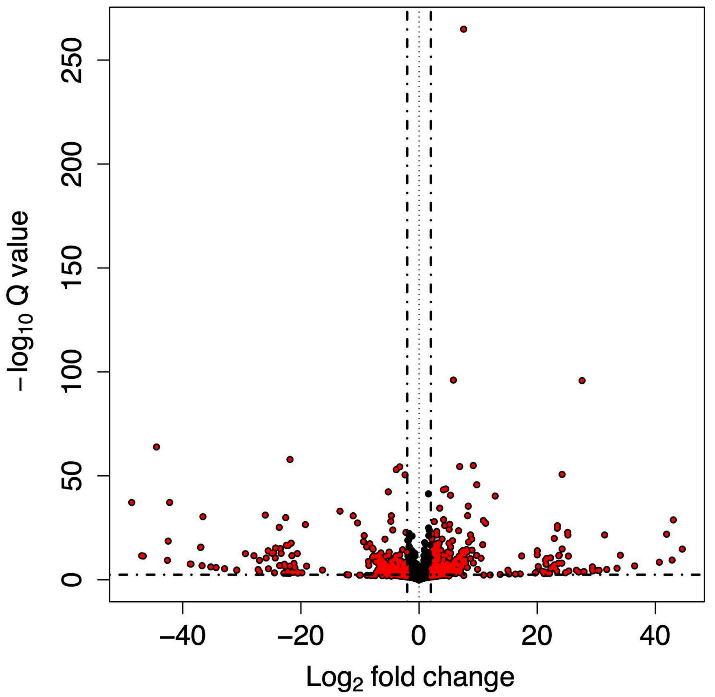

PJNo vs PJPs L

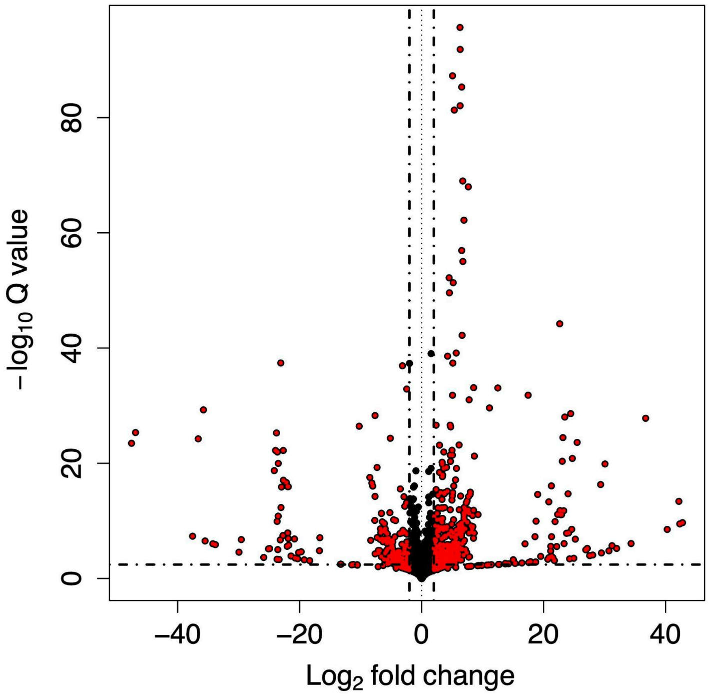

Supplement: Supplementary file 1 [file rspb.2025.3318.zip › rspb.2025.3318/rspb20253318_si_001.pdf]
