# Supplementary material for: Developmental divergence in gene regulation underlies trophic innovation in Lake Victoria cichlids
Source: Proc Biol Sci. 2026 Oct 7;293(2080):20253318. doi: 10.1098/rspb.2025.3318 (PMC13641903; doi:10.1098/rspb.2025.3318)
Supplement: Supplementary file 1 [file rspb.2025.3318.zip › rspb.2025.3318/rspb20253318_si_002.docx]

ESM-Files

Supplementary Tables:

Table 1: Number of differentially expressed genes for all species-species comparisons.

Table 2: Percentage of genes and shared direction of expression

Table 3: Number of differentially used isoforms for herbivorous versus carnivorous comparisons.

Table 4: Table of variance per species in PCA for larvae and adult samples.

Supplementary File 1: Sequencing and mapping information

Supplementary File 2:GO ids for shared differentially expressed genes in larval and adult oral and pharyngeal jaw apparatus.

Supplementary File 3: Shared differentially expressed genes (FDR ≤ 0.05) between herbivorous and carnivorous species including the direction of expression (log2FC_dir).

Supplementary File 4: Shared differentially spliced isoforms between herbivorous and carnivorous species in larval and adult oral and pharyngeal jaw apparatus.

Supplementary File 5: GO ids for shared differentially spliced isoforms in larval and adult oral and pharyngeal jaw apparatus.
